# Clinico-genomic findings, molecular docking, and mutational spectrum in an understudied population with breast cancer patients from KP, Pakistan

**DOI:** 10.3389/fgene.2024.1383284

**Published:** 2024-05-09

**Authors:** Hilal Ahmad, Asif Ali, Ali Talha Khalil, Roshan Ali, Ishaq Khan, Mah Muneer Khan, Ibrar Ahmed, Zarrin Basharat, Mohammed Alorini, Amna Mehmood

**Affiliations:** ^1^ Institute of Basic Medical Sciences (IBMS), Khyber Medical University, Peshawar, Pakistan; ^2^ Institute of Pathology and Diagnostic Medicine (IPDM), Khyber Medical University, Peshawar, Pakistan; ^3^ College of Medicine, Gulf Medical University, Ajman, United Arab Emirates; ^4^ School of Medicine, University of Glasgow, Glasgow, United Kingdom; ^5^ Department of Pathology, Lady Reading Hospital Medical Teaching Institution, Peshawar, Pakistan; ^6^ Department of Surgery, Khyber Teaching Hospital, Medical Teaching Institution, Peshawar, Pakistan; ^7^ Alpha Genomics (Private) Limited, Islamabad, Pakistan; ^8^ Microbiological Analysis Team, Group for Biometrology, The Korea Research Institute of Standards and Science (KRISS), Daejeon, Republic of Korea; ^9^ Department of Pathology, College of Medicine, Qassim University, Unaizah, Saudi Arabia; ^10^ Martin-Luther-Universität Halle-Wittenberg, Halle, Germany

**Keywords:** breast cancer, oncogenes, mutations, Pashtun, docking, clinicopathologic association, evolutionary significance

## Abstract

In this study, we report the mutational profiles, pathogenicity, and their association with different clinicopathologic and sociogenetic factors in patients with Pashtun ethnicity for the first time. A total of 19 FFPE blocks of invasive ductal carcinoma (IDC) from the Breast Cancer (BC) tissue and 6 normal FFPE blocks were analyzed by whole-exome sequencing (WES). Various somatic and germline mutations were identified in cancer-related genes, i.e., *ATM*, *CHEK2*, *PALB2*, and *XRCC2*. Among a total of 18 mutations, 14 mutations were somatic and 4 were germline. The *ATM* gene exhibited the maximum number of mutations (11/18), followed by *CHEK2* (3/18), *PALB2* (3/18), and *XRCC2* (1/18). Except one frameshift deletion, all other 17 mutations were nonsynonymous single-nucleotide variants (SNVs). SIFT prediction revealed 7/18 (38.8%) mutations as deleterious. PolyPhen-2 and MutationTaster identified 5/18 (27.7%) mutations as probably damaging and 10/18 (55.5%) mutations as disease-causing, respectively. Mutations like *PALB2 p.Q559R* (6/19; 31.5%), *XRCC2 p.R188H* (5/19; 26.31%), and *ATM p.D1853N* (4/19; 21.05%) were recurrent mutations and proposed to have a biomarker potential. The protein network prediction was performed using GeneMANIA and STRING. ISPRED-SEQ indicated three interaction site mutations which were further used for molecular dynamic simulation. An average increase in the radius of gyration was observed in all three mutated proteins revealing their perturbed folding behavior. Obtained SNVs were further correlated with various parameters related to the clinicopathological status of the tumors. Three mutation positions (*ATM* *p. D1853N*, *CHEK2 p.M314I*, and *PALB2 p.T1029S*) were found to be highly conserved. Finally, the wild- and mutant-type proteins were screened for two drugs: elagolix (DrugBank ID: DB11979) and LTS0102038 (a triterpenoid, isolated from the anticancer medicinal plant *Fagonia indica*). Comparatively, a higher number of interactions were noted for normal *ATM* with both compounds, as compared to mutants.

## Introduction

Breast cancer (BC) has emerged as the second foremost cause of mortalities related to cancers in women (Liu and Hu, 2023). Approximately 2.3 million cases of BC were reported in 2020 with 685,000 deaths. It is expected that by 2040, the BC incidence rates can increase up to 33.8% (Vidra et al., 2022). Statistically, 1 in 8 women generally have a lifetime risk of BC development (Dutta et al., 2023). Initially, BC was proposed as a single disease originating in the mammary gland. However, it is now established that BC is a complex disease with inter-tumor heterogeneity, and the heterogeneous nature has a significant impact on the progression of the disease and its treatment (Liu et al., 2022). Although the incidence of BC is on the rise all across the world, the mortalities and survival rates vary in different regions, which are attributed to changes in risk factors, hormonal profiles, environmental conditions, access to and standards of healthcare, and genetic features (Momenimovahed and Salehiniya, 2019). The survival rates in patients with BC are relatively higher in developed countries, as compared to LMIC (Ma and Jemal, 2013), and the general trend indicates that the BC malignancy is rapidly rising in low- and middle-income countries (Mubarik et al., 2023). Pakistan is among the countries with a higher incidence of BC among Asian countries, and one-ninth of women have the potential risk of developing breast cancer during their lifetime (Khan et al., 2021). According to the international report of the International Agency for Research on Cancer (IARC), in the year 2020, 25,928 (28.7%) new cases of BC in women were diagnosed in Pakistan with a cumulative death count of 13,725 (IARC International Agency for Resaerch on Cancer, 2020). Some reports have suggested an annual incidence of 90,000 cases/year annually with ∼16,000 deaths/year (Khaliq et al., 2019). The situation regarding BC is expected to further worsen as it is estimated that by 2030, the annual incidence of BC may rise up to 62% in Pakistan, with middle-aged patients being most vulnerable (Rubi et al., 2022). Previous reports have revealed that ∼ 89% of BC patients are diagnosed at a later stage due to the lack of awareness (Gulzar et al., 2019). With scarce resources, the lack of adequate screening points, financial constraints, lack of awareness, structural barriers, and a combination of various socio-economic factors like stigmatization, feminine sensitivity, and reluctance to visit male doctors are major challenges in the context of BC prevention and management (Sarwar et al., 2018; Khaliq et al., 2019).

Genetic anomalies and mutations are the hallmarks of cancer progression (Bao et al., 2019). The exploration of the oncogenes that are abnormally expressed in the progression of BC is, therefore, critical to develop effective BC therapeutic regimens by understanding the mechanistic aspects and propose evidence-based management strategies. The progression of BC is related to the sequence of mutations (somatic and germline) that eventually causes abnormal cell cycle, angiogenesis and apoptotic suppression, and abnormal cell proliferation that eventually ends up in a full-fledged malignancy (Economopoulou et al., 2015). Recently, the oncogenomic research has focused on the characterization of the driver somatic and germline mutations and reflects the association with various clinical phenotypes with an aim of strengthening the therapeutic regime selection for better treatment response (Mathioudaki et al., 2020).

Pathogenic variants of *ATM* (ataxia-telangiectasia mutated), *CHEK2* (checkpoint kinase 2), *PALB2* (partner and localizer of *BRCA2*), and *XRCC2* (X-ray repair cross-complementing 2) tumor suppressor genes (TSGs) have been associated with the development of cancers (Moslemi et al., 2021). Mutations in these genes are known to compromise their tumor suppression functions, which eventually cause cancerous conditions.

Previously, we reported the mutational signatures in *PTEN*, *PIK3CA*, and *TP53* in Pashtun ethnicity patients from KP (Ahmad et al., 2023). To date, no studies have been conducted on breast cancer-driven genes such as *ATM*, *PALB2*, *CHEK2*, and *XRCC2* reported from the Khyber Pakhtunkhwa region or Pashtun ethnicity and their potential associations with the various clinicopathologic and hormonal characteristics. A total of 19 confirmed BC patients with IDC and 6 paired adjacent normal tissues were used for NGS-WES. These mutations were analyzed using various *in silico* tools (MutationTaster, PolyPhen-2, SIFT, SAAFEQ-SEQ, ISPRED-SEQ, ConSurf, cBioPortal, PyMOL, etc.). Molecular dynamic simulations were carried out for selected wild and mutant proteins. Furthermore, we further applied molecular docking methods to study protein (wild and mutant)–drug interactions, which were visualized in BioDiscovery.

## Materials and methods

### Participants' enrollment and data collection

Patients with BC diagnosed with invasive ductal carcinoma (IDC) were enrolled in the study after obtaining informed consent from the patients/guardian. Formalin-fixed paraffin-embedded (FFPE) blocks of tumor with at least 70% tumor purity were included. Patients with secondary tumors were excluded. The enrolled patients were from major tertiary care hospitals (KTH-MTI and HMC-MTI). All participants were briefed on the aims and objectives of the research. Written informed consent was obtained, and their medical history was noted.

### Ethical approval

This study was approved by the Research Ethics Committee of Khyber Medical University, Peshawar vide: DIR/KMU-EB/VA/000651. All steps were performed according to the principles of the Helsinki Declaration.

### Collection of samples

Breast tumor biopsy samples were collected in 10% formalin and subsequently transferred to Histopathology Laboratory at KMU, Peshawar. Cancer Reporting Protocols and Guidelines, as described by the College of American Pathologists, were used for gross examination and reporting. A structured proforma was used to collect data regarding the color, general appearance, and consistency. Formalin-fixed paraffin-embedded (FFPE) tissue blocks were prepared in embedding cassettes, which were labeled accordingly. Rotary microtome was used for making 5-µm sections, which were placed on the glass slides and deparaffinized in xylene. H&E stain was applied for the assessment of IDC and immunohistochemical markers, i.e., ER (ERα ISO8430), PR (PR ISO6830), Her2/neu (Her2; A048529), and Ki-67 (IS 62630), for further assessment and characterization. Only the FFPE blocks having enriched tumor cell populations (at least 70%) confirmed using the florescence microscope were included in the study. Normal FFPE tissue blocks were taken from the adjacent normal tissues located at least 2 cm away from the site of the tumor (Li et al., 2018).

### Whole-exome sequencing (NGS-WES)

A total of 19 tumor rich FFPE blocks and 6 normal tissue FFPE blocks were sent for commercial next-generation whole-exome sequencing to Macrogen (Korea). The whole-exome sequence data were obtained using the Illumina Hi-Seq NGS platform with 151-bp paired-end reads. The SureSelect V6-(FFPE) reagent kit was used for the construction of library.

### Preparation of the sample and library

After the extraction of the genomic DNA, 1% agarose gel was used for QC on gel electrophoresis (30 min at 160V) after adding genomic DNA (10 µL). Samples with sufficient quantity, complemented by good bands on gel, were considered for the preparation of the library. First, random fragmentation was used for library preparation, which was followed by 5′- and 3′-end adapter ligation. The obtained fragments were amplified by PCR and gel-purified. Fluorescent quantification was used to quantify the genomic DNA.

### Whole-exome sequencing

The whole-exome sequencing data were obtained through Illumina Hi-Seq with reversible terminator-based technology for detecting single bases which are incorporated into DNA template strands. The raw files were supplied in the form of FASTQ format.

### Sequence analysis

The FASTQ files were assessed for QC by submitting these files to Babraham Bioinformatics (https://www.bioinformatics.babraham.ac.uk/projects/fastqc/). The sequence filers were aligned with human reference genome (hg38-UCSC) by the BWA (Burrows–Wheeler aligner) tool (Li and Durbin, 2010). The alignment files were then converted to BAM files using SAMtools and were further examined using the SAM mpileup tool. BCF tools were used for obtaining the Variant Call Format (VCF) file, which was submitted to the ANNOVAR program obtaining .csv files. RStudio was used for identifying various kinds of mutations such as single-nucleotide variants (SNVs), indels (insertion deletion), stop gain, and frameshift. The obtained mutations were then assessed through databases like MutationTaster, PolyPhen-2, and SIFT for characterizing the nature of the mutations. Later, these mutations were studied within the context of potential clinicopathologic factors and other social determinants.

### Bioinformatics-based predictions

The pathogenicity of the mutations was determined using the three most commonly used databases, i.e., MutationTaster, SIFT, and PolyPhen-2. The SAAFEQ-SEQ tool was used to determine the destabilizing SNVs (http://compbio.clemson.edu/lab/). Interaction site (IS) mutations were predicted using ISPRED-SEQ (https://ispredws.biocomp.unibo.it/sequence/). The evolutionary conservation scores were determined using the ConSurf web server (https://consurf.tau.ac.il/). The ConSurf web server includes an empirical Bayesian algorithm for conservation scores ranging from 1 to 9. The exposed and buried nature, as well as the functional and structural nature, was predicted. PROCHECK was used to generate Ramachandran plots.

### Mutation mapping and modeling

The lollipop plots were produced in the cBioPortal database by uploading the amino acid sequence, as discussed previously (Gao et al., 2013). The 3D structure of the mutant proteins *ATM*, *CHEK2*, *PALB2*, and *XRCC2* was visualized using PyMOL.

### Molecular dynamic simulations

GROMACS package 4.5 was used for MDS simulations for only selected interaction site mutations, as predicted through ISPRED-SEQ, and the results of the various parameters, particularly the radius of gyration (Rg) and root mean square deviation (RMSD), were compared for the wild-type and mutant proteins. The water molecules were removed, and the OPLS-AA/L all-atom force field was selected. Solvation was performed using an equilibrated three-point solvent model, i.e., spc216.gro. grompp was used to assemble the binary input file. The MDS was initiated with parameters like radius of gyration, RMSD, pressure, temperature, density, and potential.

### Molecular docking

Molecular docking of the proteins and their mutated forms were done with compounds from DrugBank (Wishart et al., 2018) and metabolites/compounds of *F. indica*. Estradiol benzoate (DrugBank ID: DB13953), estradiol valerate (DrugBank ID) elagolix (DrugBank ID: DB11979), and ketorolac (DrugBank ID: DB00465) implicated in either cancer or menstrual issues were obtained from DrugBank, while 16 compounds were obtained from the LOTUS database (https://lotus.naturalproducts.net/search/simple/Fagonia%20indica; retrieved 18 September 2023) for *F. indica*. The best scoring DB11979 and LTS0102038 against *ATM*, *PALB2*, *CHEK2*, and *XRCC2* proteins were used for docking and comparative analysis. Pocket identification was done using fpocket (https://github.com/Discngine/fpocket; retrieved 18 September 2023) (Le Guilloux et al., 2009), and docking was conducted using AutoDock Vina (Trott and Olson, 2010) deployed in BioExcel (Bayarri et al., 2022). Only one pocket was selected with the radius of 3–6 Å, volume range of 100–2000 Å³, and box offset of 12 Å. Affinities were recorded, and interacting residues were noted. Results of the docking were visualized in the BIOVIA Discovery Studio Visualizer (Biovia et al., 2000).

## Results

### Mutational landscape

**FIGURE 1:**
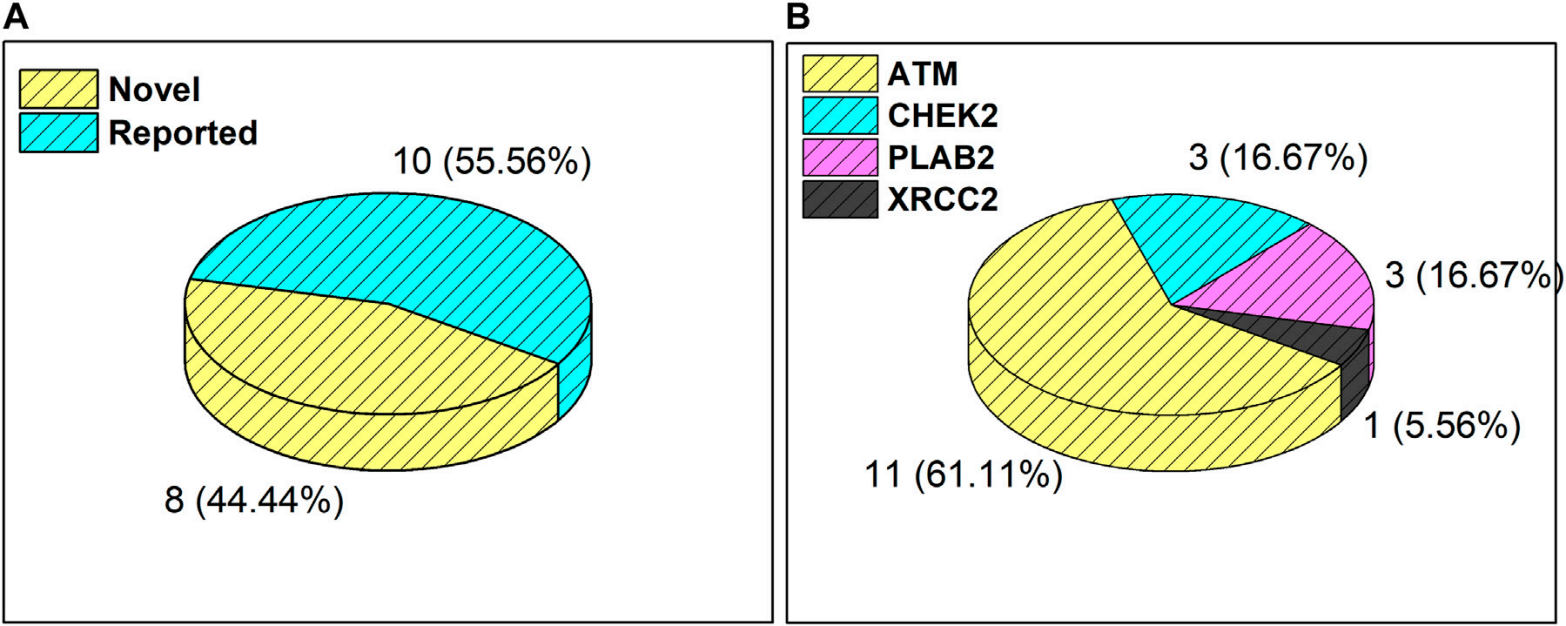
**(A)** Total number and percentage of reported and novel mutations. **(B)** Breakdown of total mutations in *ATM*, *CHEK2*, *PALB2*, and *XRCC2* genes.

**FIGURE 2:**
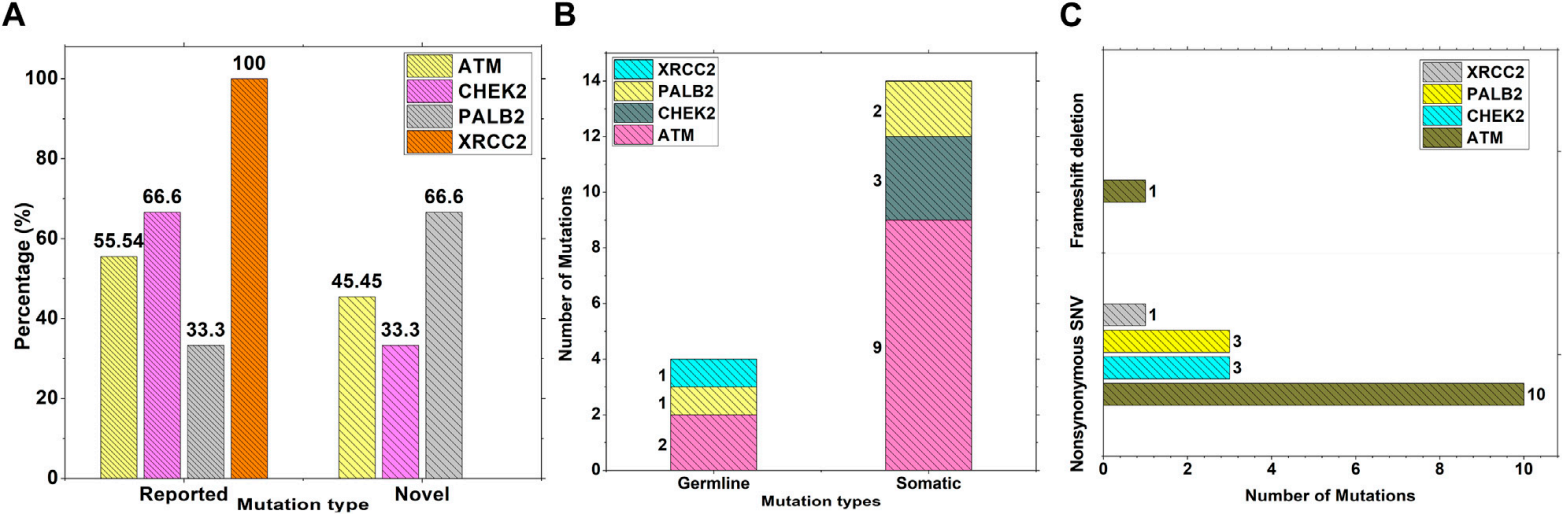
Characterization and nature of mutations on *ATM*, *CHEK2*, *PALB2*, and *XRCC2* cancer-driver genes: **(A)** Percentage of novel and reported mutations; **(B)** number of somatic and germline mutations; and **(C)** mutation types.

Results from the WES data revealed a total of 18 mutations spanning across *ATM*, *CHEK2*, *PALB2*, and *XRCC2* genes (Table 1). It was observed that 8/18 (44%) of these mutations were reported for the first time (Figure 1A). The frequency of mutations was highest in the *ATM* gene, i.e., 11/18 (61.1%). Mutations in *CHEK2*, *PALB2*, and *XRCC2* were found to be 3/18 (16.6%), 3/18 (16.6%), and 1/18 (5.5%), respectively, as depicted in Figure 1B. The inset in Figures 2A–C indicates further characterization of the obtained mutations. The total number of novel mutations in *ATM* was found to be 5/11 (45.5%). The novel reported mutations of the *ATM* gene were identified as ATM p.V1070Efs*38, *p.M1760I*, *p.W266R*, *p.S1403R*, and *p.H1568N*. For *CHEK2*, out of the three mutations, *CHEK2 p.M314I* was identified as novel, whereas two mutations *PALB2 p.I1013K* and *p.T1029S* were reported to be novel. Figure 2B reveals the number of germline and somatic mutations across *ATM, CHEK2, PALB2* and *XRCC2*. A total of 4/18 (22.2%) mutations were germline, and the rest, i.e., 14/18 (77.7%), were found to be somatic mutations. In the *ATM* gene, 2/11 (18.18%) mutations were found to be germline mutations and 9/11 (81.81%) were somatic mutations. For *CHEK2*, three somatic mutations (*CHEK2 p.R180C*, *p.M314I*, and *p.W114L*) were identified, while for *PALB2*, 2/3 mutations were somatic mutations (*PALB2 p.T1029S* and *PALB2 p.I1013K*) and 1/3 *(PALB2 p.Q559R*) was germline mutation. For *XRCC2*, the only one identified mutation was germline, i.e., *XRCC2 p.R188H*. Figure 2C reveals the nature of the mutations. The major chunk of the mutations were nonsynonymous SNVs (17/18; 94.4%), whereas only single frameshift deletion was identified in the *ATM* gene, i.e., ATM p.V1070Efs*38.

**TABLE 1:** Mutation spectrum of *ATM*, *CHEK2*, *PALB2*, and *XRCC2* genes in patients with breast cancer.

Patient code	Mutation type	Reported	Status	Location (exon)	Mutation	Mutation label	SIFT pred	PolyPhen- 2	MutationTaster
								Pred	Pred
*ATM*									
**BCR 2T, 4T**	Missense SNV	Cosmic	Somatic	Exon 37	NM_000051.4:*c*.5557G>*A*	*p.D1853N*	T	B	P
**25T**									
**27T**									
**BCR 22T**	Missense SNV	Cosmic	Somatic	Exon 41	NM_000051.4:*c*.6067G>*A*	*p.G2023R*	D	D	D
**BCR 24T**	Missense SNV	Cosmic	Somatic	Exon 20	NM_000051.4:*c*.2932T>*C*	*p.S978P*	D	D	D
**BCR 85T**	Frameshift deletion	Novel	Somatic	Exon 22	NM_000051.4:*c*.3209del*T*	p.V1070Efs*38	-	-	-
**BCR 85T**	Missense SNV	Novel	Somatic	Exon 35	NM_000051.4:*c*.5280G>*T*	*p.M1760I*	T	B	D
**BCR 114T**	Missense SNV	Novel	Somatic	Exon 7	NM_000051.4:*c*.796T>*C*	*p.W266R*	D	D	D
**BCR 114** ** ** **T/N**	Missense SNV	Cosmic	Germline	Exon 37	NM_000051.4:*c*.5630T>*C*	*p.F1877S*	T	B	N
**BCR 116T**	Missense SNV	Novel	Somatic	Exon 28	NM_000051.4:*c*.4209C>*A*	*p.S1403R*	D	D	D
**BCR 116T**	Missense SNV	Novel	Somatic	Exon 31	NM_000051.4:*c*.4702C>*A*	*p.H1568N*	T	B	N
**BCR 118T**	Missense SNV	Cosmic	Somatic	Exon 28	NM_000051.4:*c*.4138C>*T*	*p.H1380Y*	T	B	N
**BCR 120T/N**	Missense SNV	Cosmic	Germline	Exon 22	NM_000051.4: *c*3175G>*T*	*p.A1059S*	T	B	D
*CHEK2*									
**BCR 4T**	Missense SNV	Cosmic	Somatic	Exon 4	NM_007194:*c*.538C>*T*	*p.R180C*	D	P	D
**BCR 120T**	Missense SNV	Novel	Somatic	Exon 10	NM_001349956: *c*.942G>*T*	*p.M314I*	D	D	D
**BCR 120T**	Missense SNV	ClinVar	Somatic	Exon 3	NM_001349956:*c*.341G>*T*	*p.W114L*	T	B	D
*PALB2*									
**BCR 4T**	Missense SNV	Cosmic	Germline	Exon 4	NM_024675.4:*c*.1676A>*G*	*p.Q559R*	T	B	P
**24T**									
**27T**									
**85T**									
**90T/N**									
**116T/N**									
**BCR 85T**	Missense SNV	Novel	Somatic	Exon 10	NM_024675.4:*c*.3086C>*G*	*p.T1029S*	T	P	D
**BCR 118T**	Missense SNV	Novel	Somatic	Exon 10	NM_024675.4:*c*.3038T>*A*	*p.I1013K*	D	P	N
*XRCC2*									
**BCR 82T/N**	Missense SNV	Cosmic	Germline	Exon 3	NM_005431.2:*c*.563G>*A*	*p.R188H*	T	B	P
**90T/N**									
**116T/N**									
**117T/N**									
**120T/N**									

Legends: “SIFT”: D, deleterious; T, tolerated; “PolyPhen-2”: D, probably damaging; P, possibly damaging; B, benign; “Mutation Taster”: A, disease-causing automatic; D, disease causing; N, polymorphism; P, polymorphism automatic.

Note: BCR (patient enrollment code “Breast Cancer Research”); “T” for tumor FFPE block; “N” for normal FFPE block.

Details regarding the prediction from other databases such as MutationAssessor, LRT, FATHMM, PROVEAN, MetaSVM, MetaLR, and M-CAP are provided in Supplementary Table S2.

**FIGURE 3:**
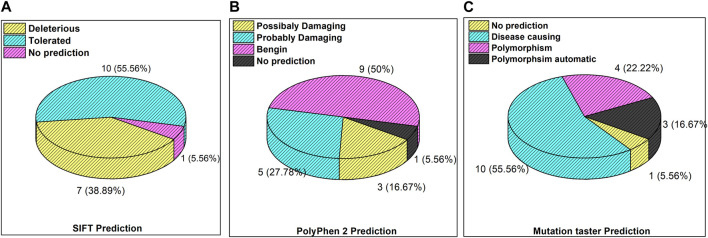
Mutation predictions from different databases in the enrolled cohort: **(A)** SIFT prediction; **(B)** PolyPhen-2 prediction; and **(C)** MutationTaster prediction.

The inset in Figures 3A–C reveals the overall prediction of the obtained mutations which were assessed using different databases like SIFT, PolyPhen-2, and MutationTaster. Overall, 7/18 (38.8%) predications were made by SIFT as deleterious, while 1/18 mutations, i.e., ATM p.V1070Efs*38, have no prediction SIFT, PolyPhen-2 and Mutation Taster. The prediction from PolyPhen-2 databases revealed 5/18 (27.7%) and 3/18 (16.6%) mutations as probably damaging and possibly damaging, respectively. The MutationTaster database revealed that 10/18 (55%) mutations is disease-causing. Further predictions related to the mutation pathogenicity such as FATHMM and PROVEAN are supplied, as depicted in Supplementary Table S2.

Supplementary Figure S4 (A–C) depicts the gene wise prediction of the pathogenic mutations. The SIFT prediction for *ATM, CHEK2, PALB2* revealed a total of 4/11, 2/3 and 1/3 mutations as deleterious respectively. The Poly–Phen 2 prediction for ATM revealed 4/11 as possibly damaging and 6/11 benign mutations. For *PALB2*, 2/3 mutations were identified as possibly damaging. Mutation taster revealed 6/11, 3/3 and 1/3 disease causing mutations in *ATM, CHEK2* and *PALB2* respectively.

**FIGURE 4:**
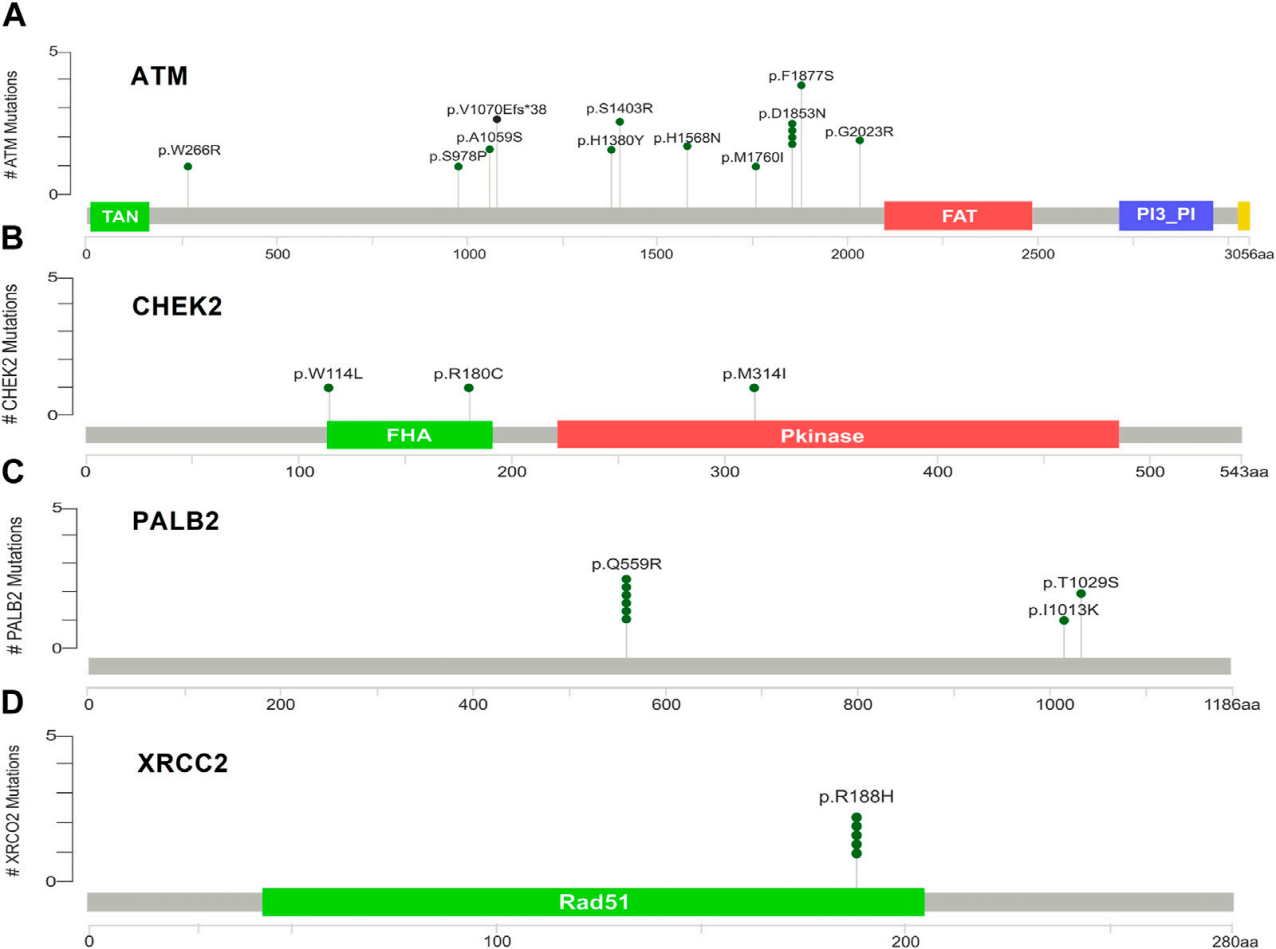
Lollipop plot of mutations on **(A)** *ATM*; **(B)** *CHEK2*; **(C)** *PALB2*; and **(D)** *XRCC2*. cBioPortal was used to obtain these plots.

Supplementary Figures S5A–D indicate the distribution of mutations on exons. For *ATM*, two mutations each were found to be located on exon 22, exon 28, and exon 37. In *CHEK2*, one mutation each was identified on exon 3, exon 4, and exon 10, whereas, for *PALB2*, two mutations were found on exon 10 and 1 mutation on exon 4. For *XRCC2*, only one mutation was identified located on exon 3. The lollipop plot acquired from the cBioPortal is depicted in Figures 4A–D, which shows the frequency of mutations in the enrolled cohort. Mutations like *PALB2 p.Q559R* (6/19; 31.5%), *XRCC2 p.R188H* (5/19; 26.31%), and *ATM p.D1853N* (4/19; 21.05%) were found to be recurring in the enrolled cohort (Supplementary Figure S7) and hence have a biomarker potential.

**FIGURE 5:**
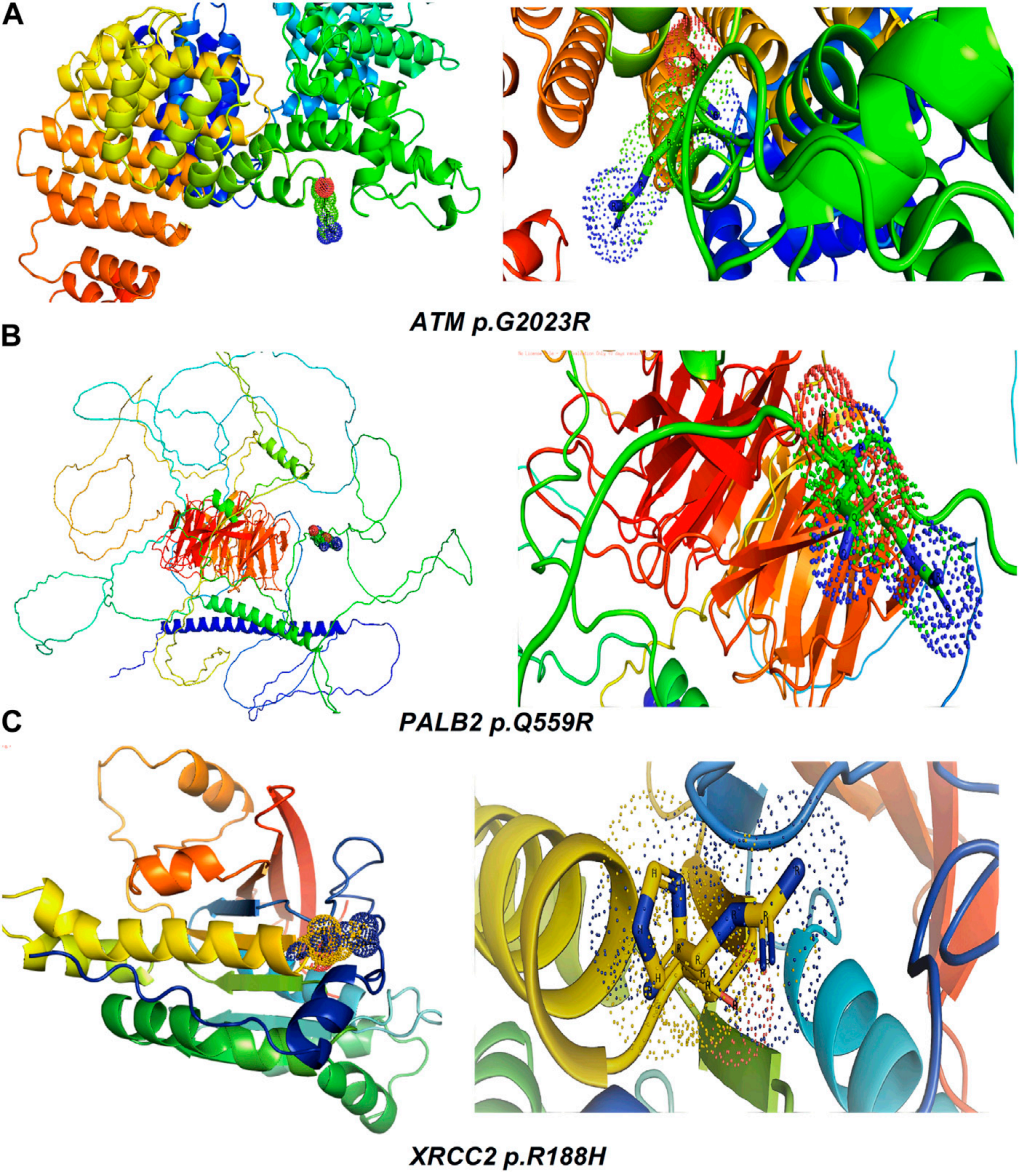
Visualization and superimposition of interaction site mutations in PyMOL: **(A)** *ATM p.G2023R*; **(B)** *PALB2 p.Q559R*; **(C)** *XRCC2 p.R188H*.

Table 2 reveals the SAAFEQ-SEQ predictions that relate to the effect of the SNVs on protein stability. The algorithm is based on various parameters that compute the change in stability-free energy resulting from SNVs. The destabilizing effect was predicted for all obtained SNVs. To further scrutinize these mutations, we applied the ISPRED-SEQ tool for identifying only the interaction site (IS) mutations, and the results are summarized in Table 3. Only three IS mutations were identified one each on *ATM p.G2023R*, *PALB2 p.Q559R*, and *XRCC2 p.R188H*, whereas no IS mutations were identified on *CHECK2*. The IS mutations were superimposed and visualized in PyMOL, as depicted in Figures 5A–C, and later further evaluated for MDS simulation to identify the differences in the wild-type and mutant proteins in terms of various parameters, especially radius of gyration (Rg) and root mean square deviation (RMSD), as revealed in Supplementary Figures S9A–H, Supplementary Figures S10A–H, and Supplementary Figures S11A–H.

**TABLE 2:** SAAFEC-SEQ predictions for protein stability (https://ispredws.biocomp.unibo.it/sequence/).

Protein	Mutation label	ddG	Effect on protein
*ATM*	*p.D1853N*	−0.21	Destabilizing
	*p.G2023R*	−0.36	Destabilizing
	*p.S978P*	−0.15	Destabilizing
	*p.M1760I*	−0.60	Destabilizing
	*p.W266R*	−1.77	Destabilizing
	*p.F1877S*	−1.37	Destabilizing
	*p.S1403R*	−1.02	Destabilizing
	*p.H1568N*	−0.43	Destabilizing
	*p.H1380Y*	−0.24	Destabilizing
	*p.A1059S*	−1.13	Destabilizing
*CHEK2*	*p.R180C*	−0.81	Destabilizing
	*p.M314I*	−1.06	Destabilizing
	*p.W114L*	−1.05	Destabilizing
*PALB2*	*p.Q559R*	−0.28	Destabilizing
	*p.T1029S*	−0.17	Destabilizing
	*p.I1013K*	−1.33	Destabilizing
*XRCC2*	*p.R188H*	−0.23	Destabilizing

**TABLE 3:** ISPRED-SEQ predictions for determining interaction sites.

S. No.	Mutation label	Status with probability
1	*XRCC2 p.R188H*	IS (0.54)
2	*PALB2 p.I1013K*	NIS
3	*PALB2 p.Q559R*	IS (0.68)
4	*PALB2 p.T1029S*	NIS
5	*CHEK2 p.M314I*	NIS
6	*CHEK2 p.R180C*	NIS
7	*CHEK2 p.W114L*	NIS
8	*ATM p.G2023R*	IS (0.63)
9	*ATM p.A1059S*	NIS
10	*ATM p.D1853N*	NIS
11	*ATM p.F1403R*	NIS
12	*ATM p.F1877S*	NIS
13	*ATM p.H1380Y*	NIS
14	*ATM p.H1568N*	NIS
15	*ATM p.M1760I*	NIS
16	*ATM p.S978P*	NIS
17	*ATM p.W266R*	NIS

Note: All mutations of PTEN were non-interacting.

**FIGURE 6:**
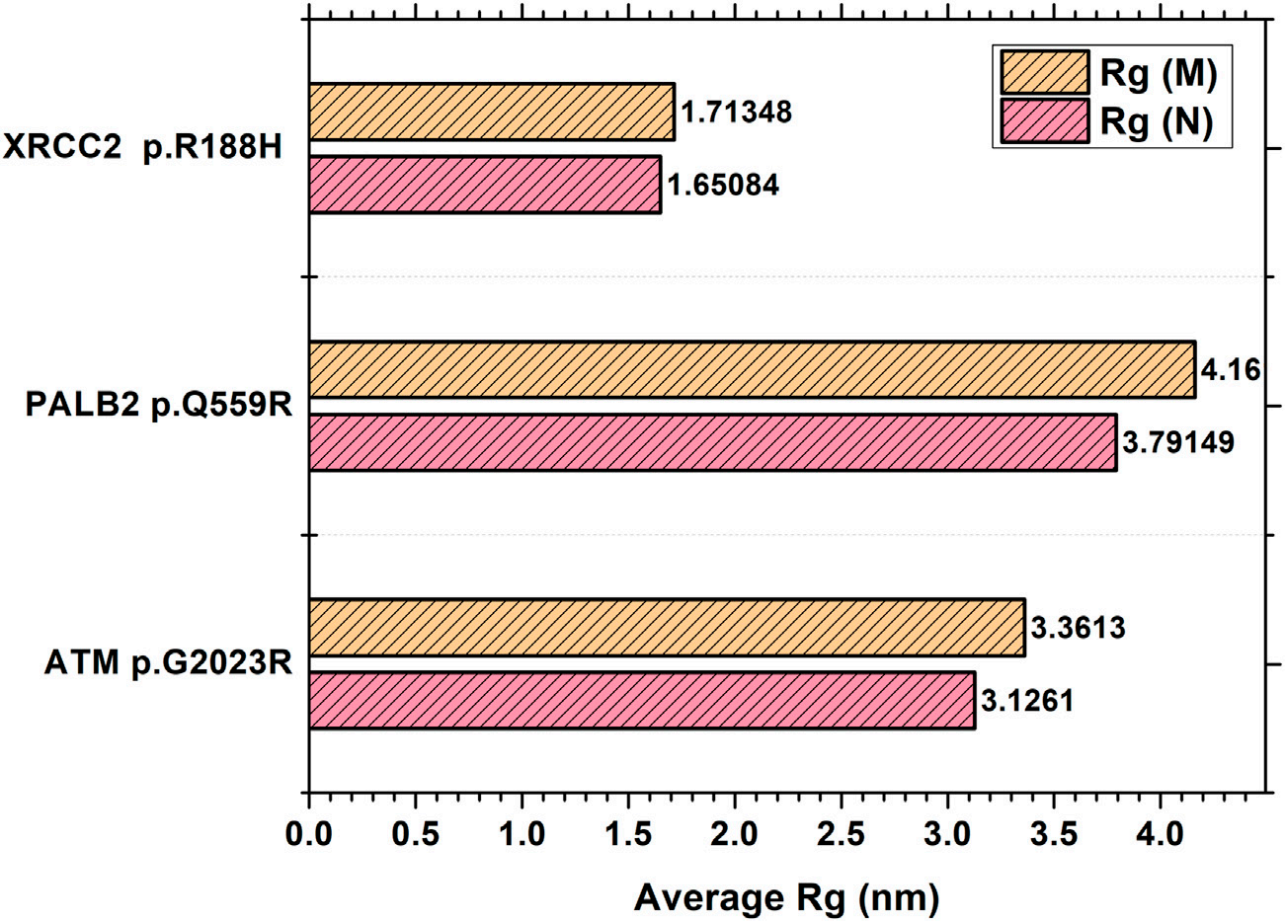
Average radius of gyration for the selected interaction site mutations.

The MDS simulation results of *ATM p.G2023R* revealed an average Rg of 3.126 nm and 3.36 nm for the wild and mutant type, respectively. Similarly, the average Rg for *PALB2 p.Q559R* was reported to be 3.79 nm (wild type) and 4.16 nm (mutant type), whereas for *XRCC2 p.R188H*, the average Rg was recorded as 1.65 nm (wild) and 1.713 nm (mutant), as depicted in Figure 6. RMSD values were calculated for the normal and mutant proteins for determining the overall changes to the stability of protein. For *ATM p.G2023R*, the RMSD simulation revealed structural differences starting from ∼0.4 ns. Across the simulation, the wild-type and mutant *ATM* showed minor and major deviations. Similarly, for *PALB2 p.Q559R*, the structural deviations were observed, which started approximately ∼ 1.3 ns and remained deviated along the course of trajectory. For *XRCC2 p.R188H*, major structural deviations were noted during the simulation, which started around 1.4 ns. Other MDS simulation results are depicted in the inset in Supplementary Figures S9A–H, Supplementary Figures S10A–H, and Supplementary Figures S11A–H, whereas the corresponding Ramachandran plots for the wild-type and mutant proteins are also indicated. The major difference was observed only in *ATM p.G2023R*, in which the residues in the favorable region for the wild type (93.4%) were decreased (93.2%) in the mutant type. For *PALB2 p.Q559R* and *XRCC2 p.R188H*, no differences were observed.

Table 4 summarizes the results obtained from the ConSurf tool, which relates to the evolutionary conservation of the residue position based on its structural and functional importance. Among the obtained SNVs, three mutations, i.e., *ATM p.D1853N, CHEK2* *p.M314I,* and *PALB2* *p.T1029S* were found to be highly conserved (ConSurf score: 08), and among them, *ATM p.D1853N* was of functional importance. *ATM p.S978P, ATM p.G2023R*, and *PALB2* *p.I1013K* were also identified as a conserved position (ConSurf score: 07).

**TABLE 4:** ConSurf prediction of the SNVs of *ATM*, *CHEK2*, *PALB2*, and *XRCC2*.

S. No.	*ATM*			
	Mutation label	Conservation scores (1–9)	Finding	Screenshot from the ConSurf prediction
1	*ATM p.W266R*	6	Buried and moderately conserved	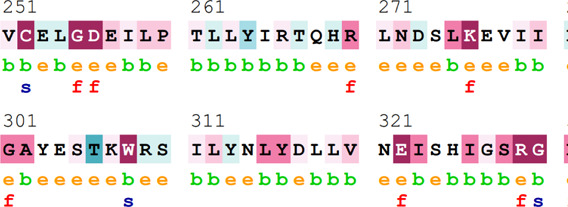
2	*ATM p.S978P*	7	Exposed and moderately conserved	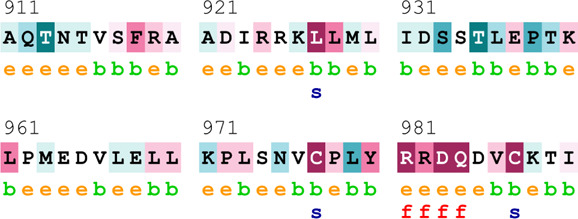
3	*ATM p.A1059S*	6	Buried and moderately conserved	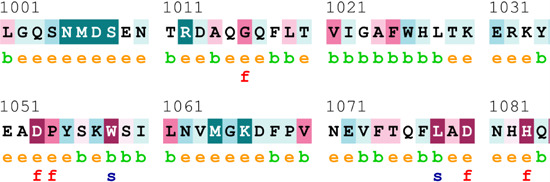
4	*ATM p.H1380Y*	4	Average and exposed	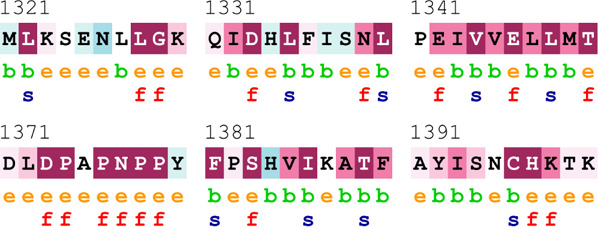
5	*ATM p.F1403R*	6	Exposed and moderately conserved	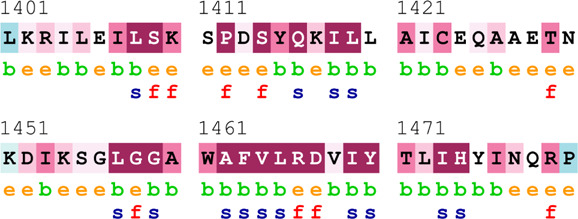
6	*ATM p.H1568N*	3	Exposed and variable	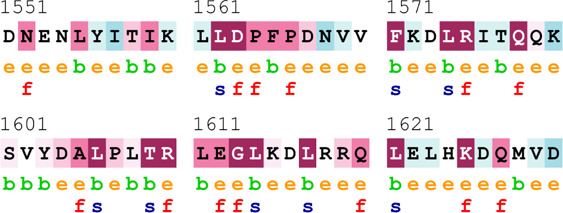
7	*ATM p.M1760I*	6	Buried and moderately conserved	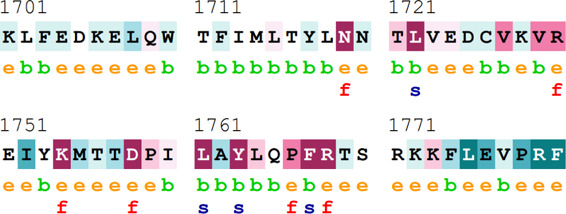
***8***	*ATM p.D1853N*	8	Conserved and functional	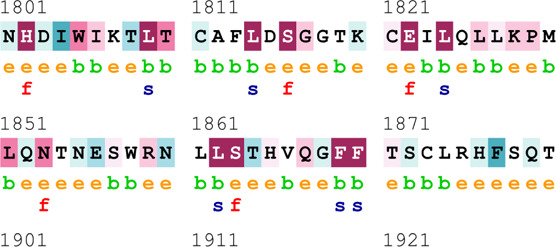
***9***	*ATM p.F1877S*	4	Average and exposed	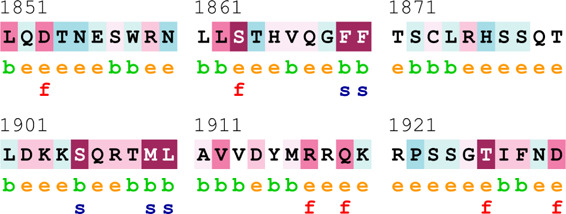
***10***	*ATM p.G2023R*	7	Exposed and moderately conserved	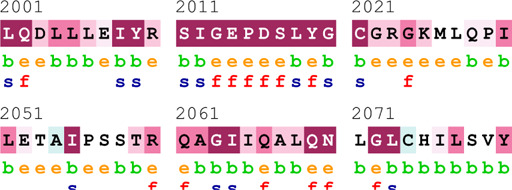
*CHEK2*				
11	*CHEK2 p.W114L*	6	Exposed and moderately conserved	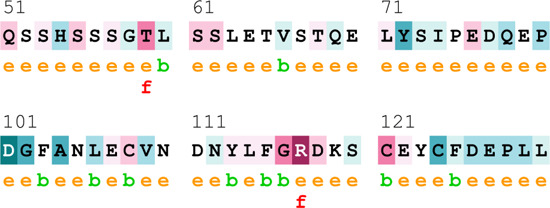
12	*CHEK2 p.R180C*	5	Exposed	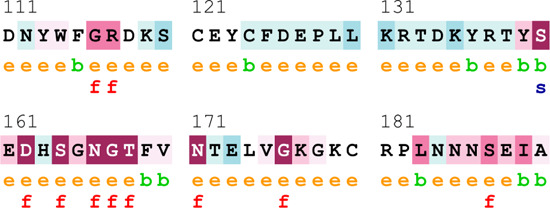
13	*CHEK2 p.M314I*	8	Conserved and buried	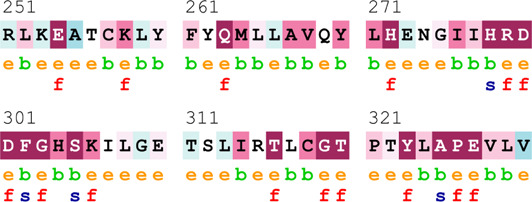
*PALB2*				
14	*PALB2 p.Q559R*	4	Average and exposed	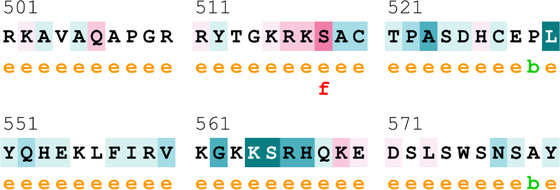
15	*PALB2 p.I1013K*	7	Buried and conserved	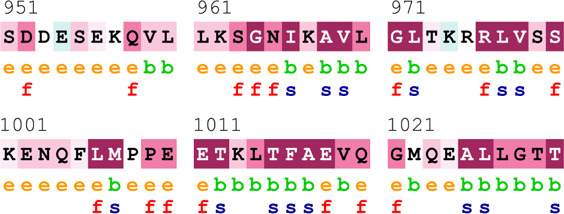
16	*PALB2 p.T1029S*	8	Conserved and buried	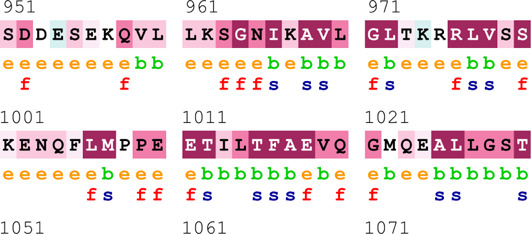
*XRCC2*				
17	*XRCC2 p.R188H*	5	Buried	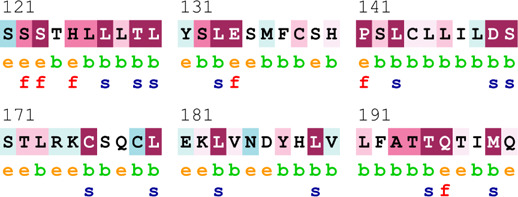
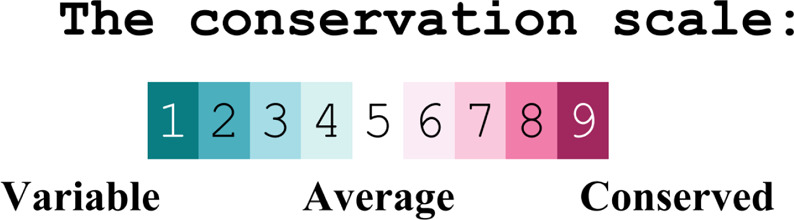				

### Clinicopathologic association

The inset in Supplementary Figures S12A,B further characterizes the specific *ATM* mutations. The mutation *ATM p.D1853N* was found to be present in grade 2 and grade 3 tumors. *ATM p.S978P* was the only *ATM* mutation present in Her2+, while all the rest of the mutations were only present in Her2−. Three *ATM* mutations (*ATM p.H1380Y*, *p.S1403R*, *and p.H1568N*) were found to be associated only with triple negative molecular subtypes. The inset in Supplementary Figures S13A–C summarizes the immunohistochemical association of the mutations in *CHEK2*, *PALB2*, and *XRCC2*. It is evident from the results that *CHEK2 p.R180C*, *p.M314I*, and *p.W114L* were not identified in ER− and Her2+. *CHEK2 p.R180C* was found only in PR+, and the rest *CHEK2 p.M314I* and *p.W114L* were only found in PR−. *PALB2 p.Q559R* was found to be distributed in all major categories, i.e., ER+, ER−, PR+, PR−, Her2+, and Her2, as depicted in Supplementary Figures S13A–C. Similarly, *PALB2 p.Q559R* was found to be associated with Her+, triple negative, and Luminal A and Luminal B molecular subtypes. The same mutation, i.e., *PALB2 p.Q559R*, was found both in grade 2 and grade 3 tumors, as depicted in Supplementary Figure S14B. The *PALB2 p.I1013K* mutation was only found in the triple-negative breast cancer patients, as shown in Supplementary Figure S14B. *XRCC2 p.R188H* was found in grade 2 and grade 3 tumors. Similarly, *XRCC2 p.R188H* was found in ER+, ER−, PR+, PR−, Her2+, and Her2− patients and was also identified in Luminal A, Luminal B, and triple-negative molecular subtypes.

### Docking of *ATM*, *CHEK2*, *PALB2*, and *XRCC2* mutants

Molecular docking was done for both the normal and mutated proteins with an FDA-approved drug elagolix (DrugBank databse ID: DB11979) and a triterpenoid saponin (IUPAC name: (2S,3R,4S,5S,6R)-3,4,5-trihydroxy-6-(hydroxymethyl)oxan-2-yl (1S,2R,4aS,6aS,6bR,8aR,10S,12aR,12bR,14bS)-10-{[(2S,3R,4S, 5S)-5-hydroxy-3 {[(2S,3R,4S,5S,6R)-3,4,5-trihydroxy-6-(hydroxymethyl)oxan-2-yl]oxy}-4-{[(2S,3R,4S,5S) 3,4,5-trihydroxy oxan-2-yl]oxy}oxan-2-yl]oxy}-1,2,6a,6b,9,9,12a-heptamethyl 1,2,3,4,4a,5,6,6a,6b,7,8,8a,9,10,11,12,12a,12b,13,14b-icosahydropicene-4a-carboxylate) (LOTUS database ID: LTS0102038) from *F. indica* for comparison (Table 5). A higher number of interactions were observed for normal *ATM* with both compounds, compared to mutants. Around 14 residues were conserved in an interaction with normal proteins, while the residues were themselves changed for a mutant interaction compared to the normal protein. Overall, the binding affinity was better for the F1877S mutant of *ATM*, compared to the normal protein. The binding affinity of these compounds was good for normal *ATM p.G2023R* but low for the mutant *ATM p.G2023R*. The *ATM p.W266R* did not show good affinity in the normal or mutated state (*ATM p.W266R*), as well as XRCC p.R188H. PALB2 showed good binding affinity in both normal and mutated states. *ATM* mutants and *PALB2* normal and mutants, showing good binding affinities for the studied compounds may be explored further through other *in silico*, *in vitro*, and *in vivo* assays.

**TABLE 5:** Details of the interactions of proteins and their mutants with DB11979 and LTS0102038. Histidine with hydrogen on epsilon nitrogen is mentioned as Hie.

Protein	Compound	Affinity score	Binding residue	Interaction	2D visualization
***ATM F1877S mutant***	DB11979	−7.0	Asn96, Glu93, Ser98, Ser123, Lys127, Hie176, Hie183, Leu184, Ser187, Lys191, Arg229, Leu233, Asp236, and Lys180	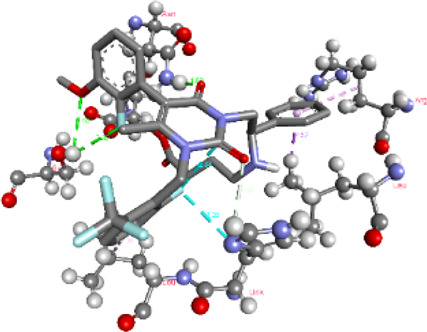	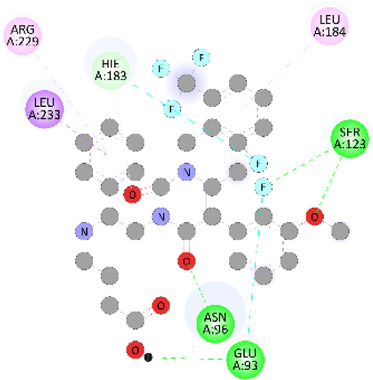
***ATM F1877S mutant***	LTS0102038	−6.5	Asn217, Gln218, Arg219, Gly262, Ile265, Pro266, Tyr269, Pro306, Asp307, Hie308, Thr349, Leu351, Glu352, Lys355, Thr402, Gly403, and Glu406	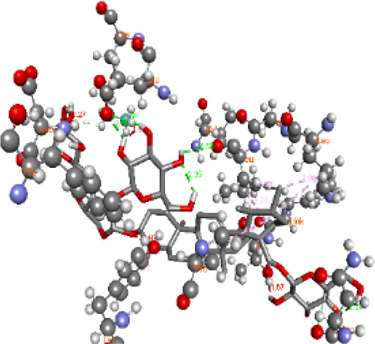	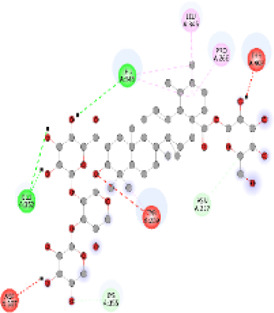
***ATM F1877 normal***	DB11979	17.7	Gln917, Gly920, Glu921, Ser924, Ile925, Glu927, Leu928, Phe929, Arg931, Val933, Gln937, Leu938, Glu940, Val941, Tyr942, Lys944, Trp945, Ile970, Leu971, Leu974, Glu978, Ile987, and Ile990	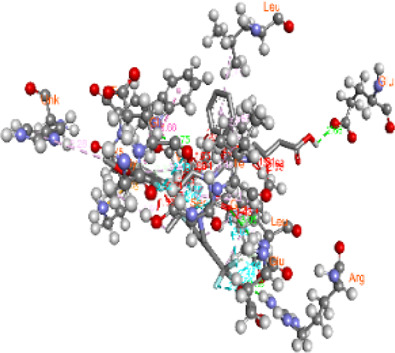	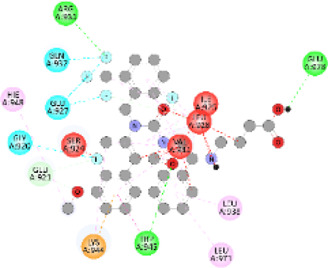
***ATM F1877 normal***	LTS0102038	55.8	Glu921, Ser924, Ile925, Glu927, Leu928, Phe929, Leu938, Glu940, Val941, Lys944, Trp945, Glu978, Asp980, Gln983, Arg931, Ser932, Val933, Thr934, Hie935, Gln937, Ile970, Leu971, Leu974, Lys977, Arg878, Ile987, and Ile990	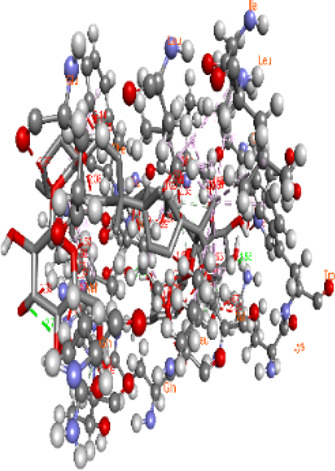	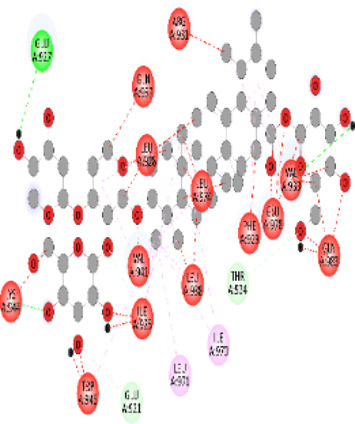
***ATM G2023R mutant***	DB11979	−5.4	Ala12, Trp5, Leu9, Phe13, Ser16, Leu42, Ile46, Trp58, Leu61, Leu62, Hie65, Val66, phe69, Hie95, Arg98, Met109, and Val112	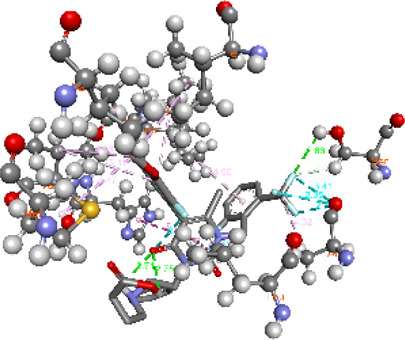	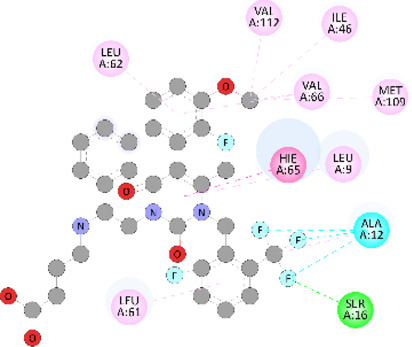
***ATM G2023R mutant***	LTS0102038	11.7	Trp5, Leu9, Ala12, Phe13, Ser16, Ile46, Trp58, Leu61, Leu62, Hie65, Val66, Phe69, Phe70, Arg98, Met109, Val112, Val113, Met116, Leu134, and Leu42	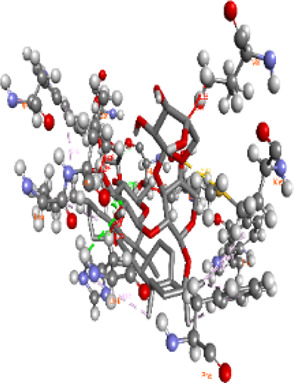	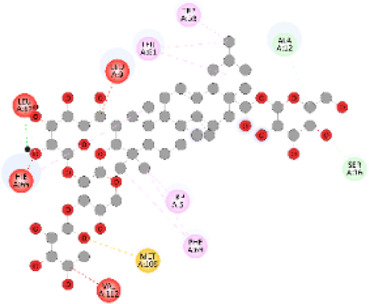
***ATM G2023 normal***	DB11979	−5.0	Tyr344, Lys348, Arg351, Val352, Val355, Glu356, Leu376, and Glu383	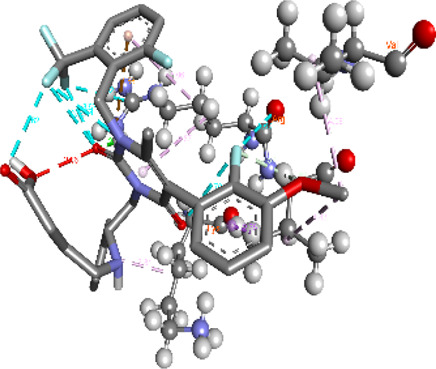	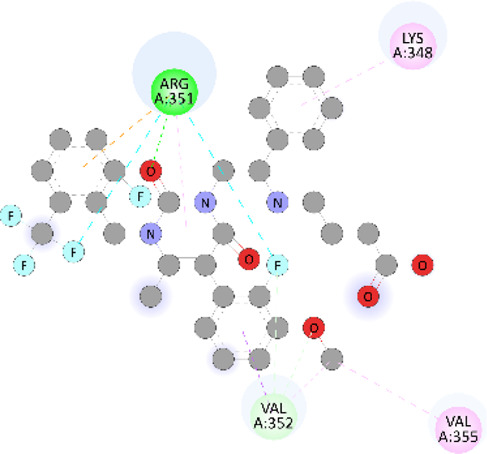
***ATM G2023 normal***	LTS0102038	−5.5	Gly321, Lys348, Tyr349, Arg351, Val352, Lys353, Val355, Glu356, Glu357, and Lys360	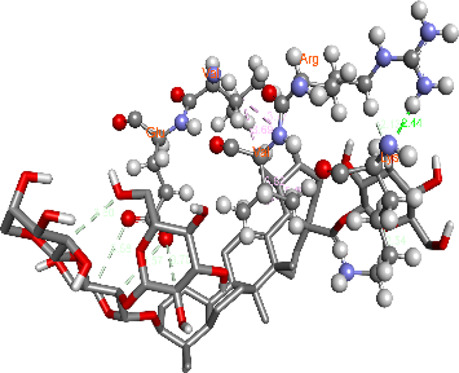	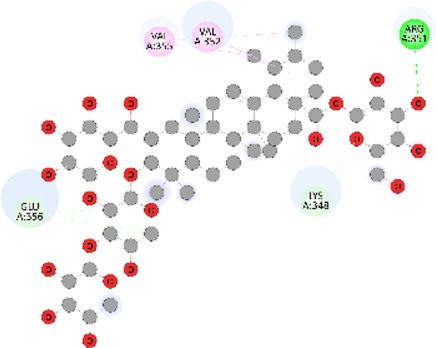
***ATM W266R mutant***	DB11979	9.2	Leu970, Leu973, Ser974, Cys977, Ser978, Arg981, Gln1017, Thr1020, Val1021, Ala1024, and Leu1028	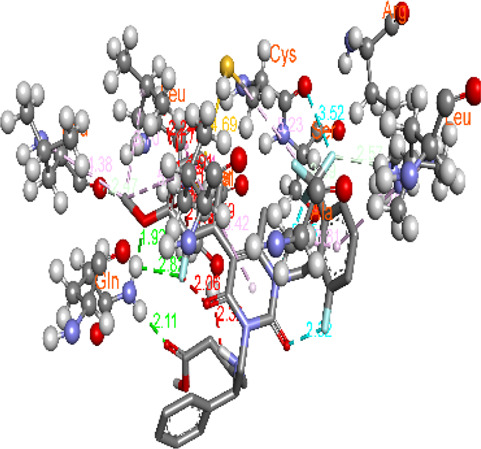	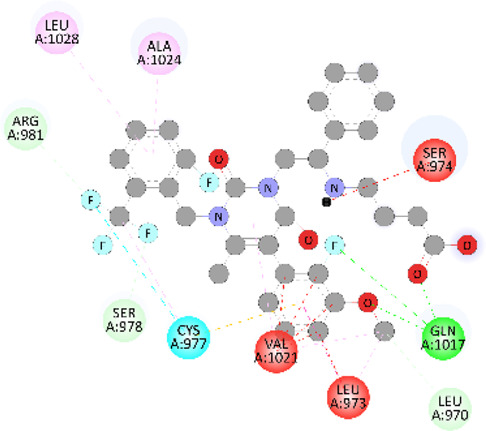
***ATM W266R mutant***	LTS0102038	42.3	Leu970, Lys971, Pro972, Leu973, Ser974, Asn975, Val976, Cys977, Ser978, Tyr980, Arg981, Arg982, Asp983, Val986, Cys987, Lys988, Thr989, Ile990, Leu991, Gln1017, Thr1020, Val1021, Ala1024, Phe1025, Leu1028, and Tyr1034	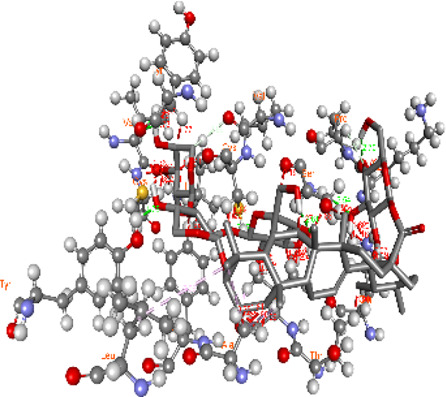	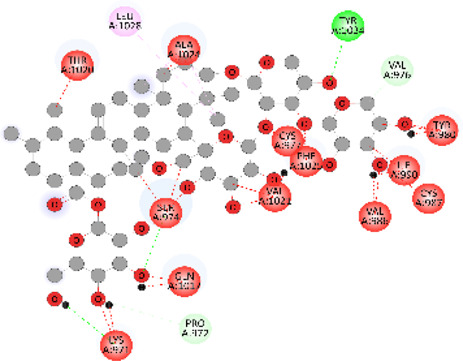
***ATM W266 normal***	DB11979	3.2	Ser974, Cys977, Ser978, Arg981, Gln1017, Thr1020, Val1021, Ala1024, Phe1025, Leu1028, and Tyr1034	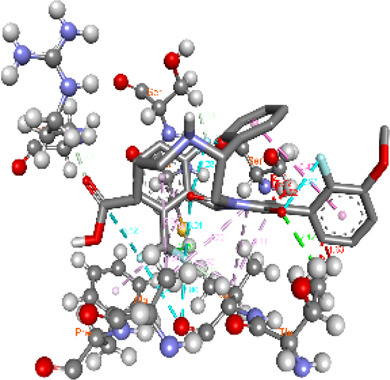	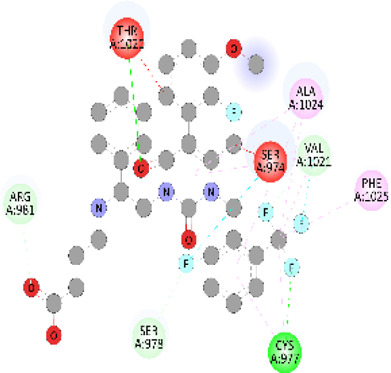
***ATM W266 normal***	LTS0102038	42	Tyr947, Leu948, Arg981, Leu951, Leu970, Lys971, Pro972, Leu973, Ser974, Asn975, Val976, Cys977, Ser978, Cys987, Ile990, Leu991, Val994, Gln1017, Phe1018, Thr1020, Val1021, Ile1022, Ala1024, Phe1025, Leu1028,Tyr1034, and Cys1045	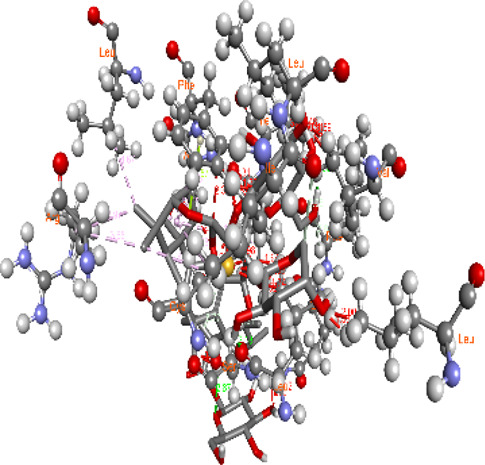	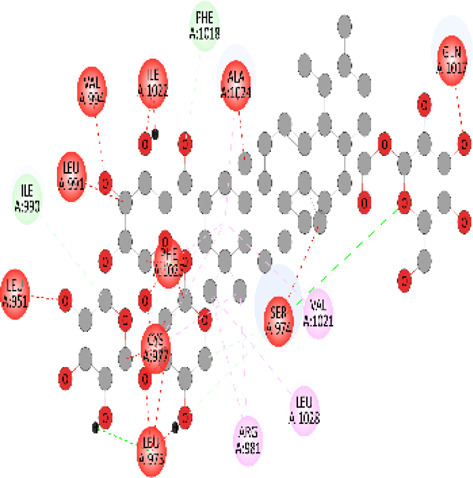
***PALB2 Q559R mutant***	DB11979	−6.7	Phe404, Pro405, Tyr408, Arg411, Thr412, Ser415, Met416, Ala727, Pro729, Ile730, Leu731, Gly732, Lys967, Ile1013, Leu1014, Thr1015, Ile1031, Ala1057, Val1059, Pro1077, Cys1078, Phe1118, Leu1119, and Thr1133	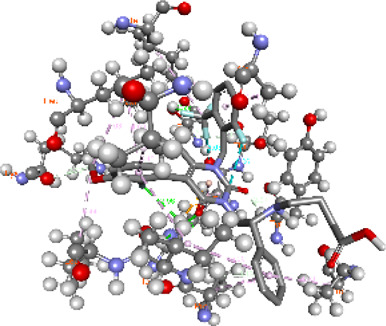	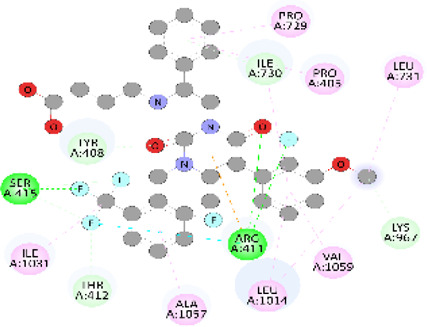
***PALB2 Q559R mutant***	LTS0102038	−3.3	Leu403, Phe404, Pro405, Glu407, Tyr408, Arg411, Thr412, Cys724, Pro726, Ala727, Pro729, Ile730, Leu731, Gly732, Ser725, Lys967, Ala968, Leu1014, Thr1015, Ala1057, Val1059, Pro1077, Cys1078, Arg1117, Phe1118, Leu1119, Thr1133, and Lys1176	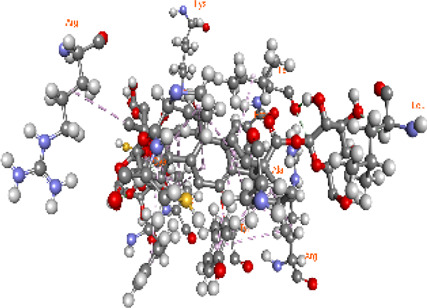	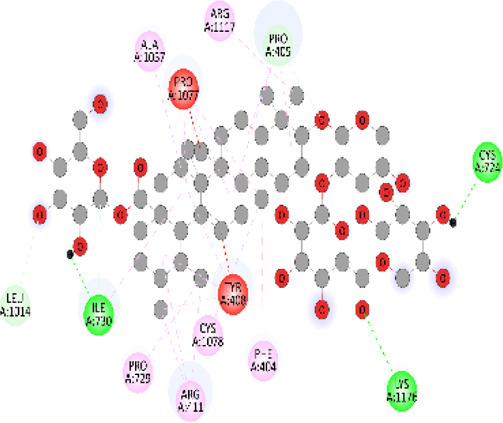
***PALB2 Q559R normal***	DB11979	−7.2	Phe404, Pro405, Tyr408, Arg411, Thr412, Ser415, Met416, Pro729, Ile730, Leu731, Leu939, Glu940, Lys967, Ile1013, Leu1014, Ile1031, Ala1057, Pro1077, Cys1078, Phe1118, and Leu1119	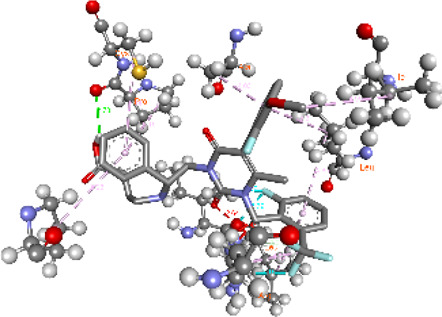	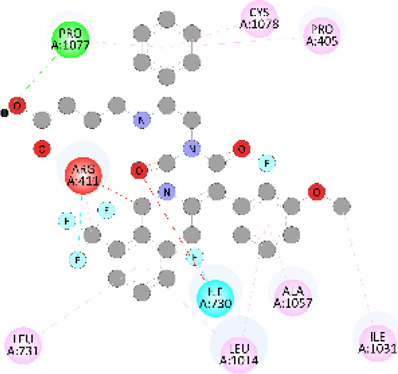
***PALB2 Q559R normal***	LTS0102038	−2.7	Leu403, Phe404, Pro405, Glu407, Tyr408, Arg411, Thr412, Cys724, Ser725, Pro726, Ala727, Pro729, Ile730, Leu731, Gly732, Lys967, Leu1014, Thr1015, Ala1057, Val1059, Arg1117, Phe1118, Leu1119, Thr1133, Lys1176, Pro1077, and Cys1078	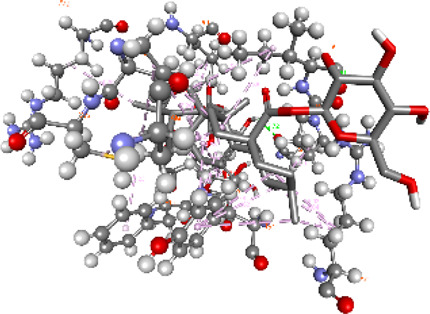	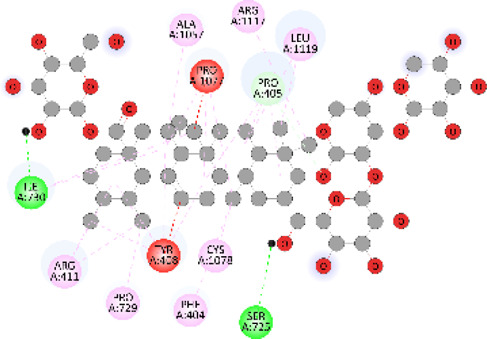
***XRCC R188H mutant***	DB11979	29.8	Thr83, Ser123, Thr124, Leu126, Leu130, Leu148, Ser150, Leu151, Ser152, Ala153, Phe154, Tyr155, Asp158, Arg159, Asn161, Gly162, Leu168, Gln169, Glu170, Ser171, Thr172, Leu173, Cys176, Ser177, Leu180, Tyr226, Leu227, and Cys228	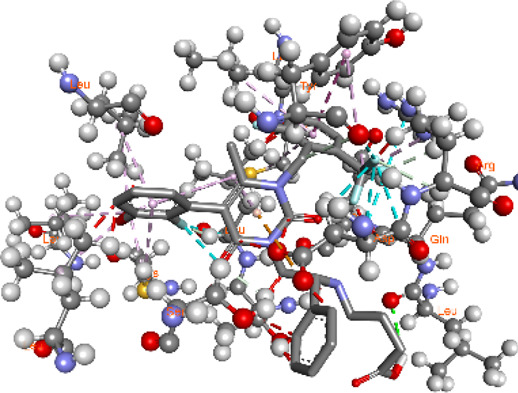	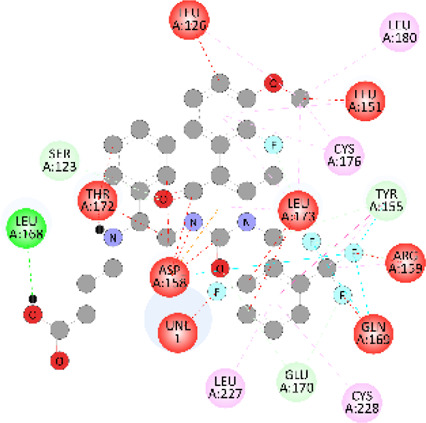
***XRCC R188H mutant***	LTS0102038	129.2	Ser122, Ser123, Thr124, Leu126, Leu127, Leu130, Leu148, Ser150, Leu151, Ser152, Ala153, Phe154, Tyr155, Asp158, Arg159, Asn161, Leu168, Gln169, Glu170, Ser171, Thr172, Leu173, Arg174, Cys176, Ser177, Leu180, Leu191, Pro225, Tyr226, Leu227, Cys228, Lys229, Ala230, Trp231, and Gln232	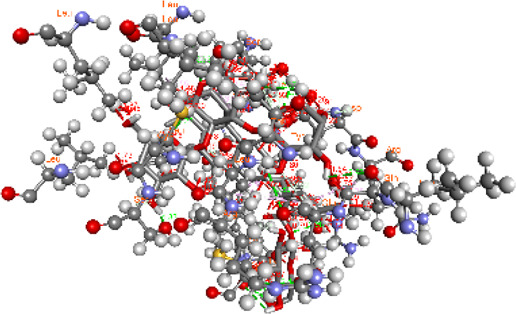	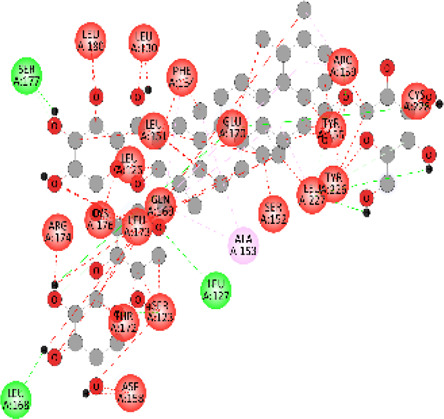
***XRCC R188H normal***	DB11979	26.2	Thr83, Ser123, Thr124, Leu126, Leu148, Ser150, Leu151, Ser152, Phe154, Tyr155, Asp158, Arg159, Asn161, Leu168, Gln169, Glu170, Ser171, Thr172, Leu173, Cys176, Tyr226, Leu227, and Cys228	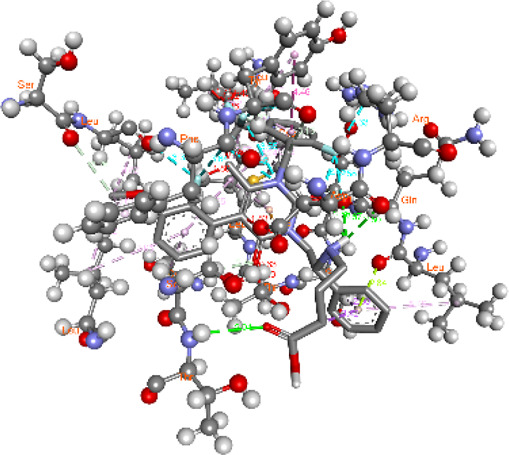	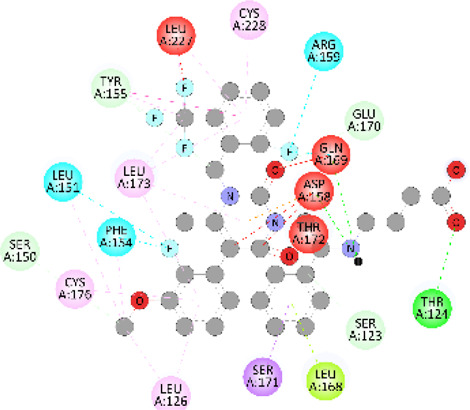
***XRCC R188H normal***	LTS0102038	125.1	Thr83, Leu180, Pro225, Tyr226, Leu227, Cys228, Lys229, Trp231, Gln232, Leu148, Ser150, Leu151, Ser152, Ala153, Tyr155, Phe154, Asp158, Arg159, Ser122, Ser123, Leu126, Leu127, Leu130, Leu168, Gln169, Glu170, Ser171, Thr172, Leu173, Arg174, Cys176, Ser177, and Met199	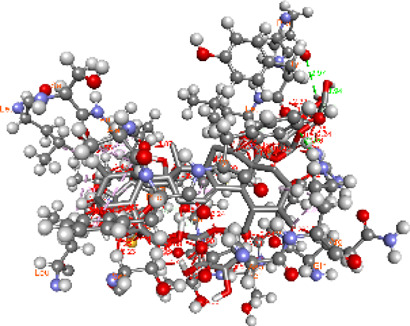	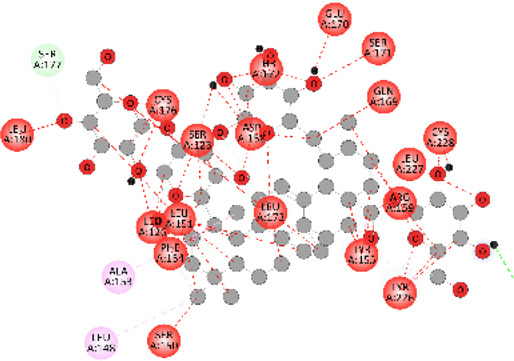

## Discussion

**FIGURE 7:**
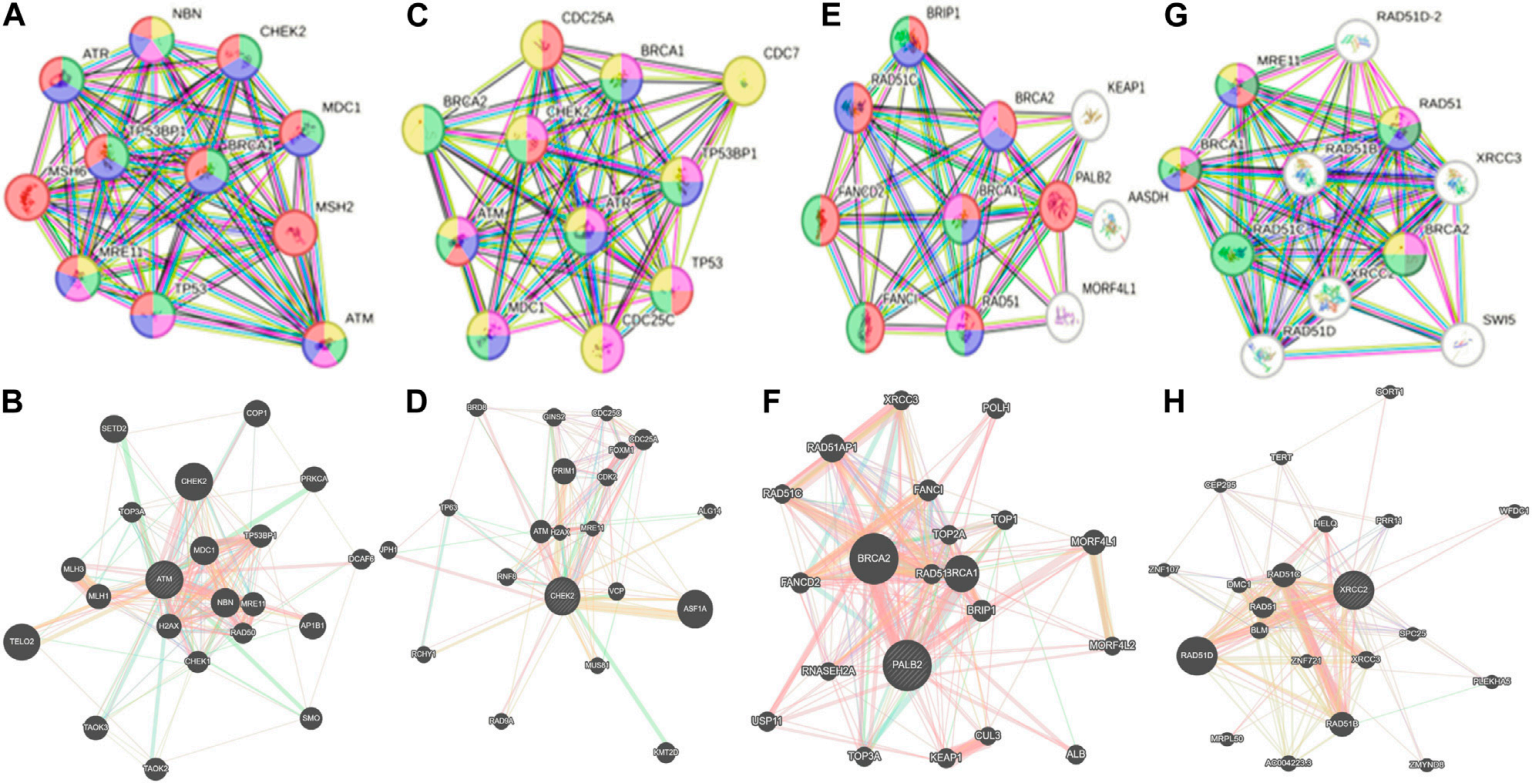
Various interaction networks of candidate genes retrieved from STRING and GeneMANIA; **(A,B)** *ATM*; **(C,D)** *CHEK2*; **(E,F)** *PALB2*, and **(G,H)** *XRCC2*.

Whole-exome sequencing is now considered an important tool for screening the somatic and germline mutations, which are of particular interest in cancer. Pathogenic mutations in cancer-driven genes like *ATM*, *CHEK2*, *PALB2*, and *XRCC2* have been reported to increase the risk of different malignancies, especially breast and prostate cancers. These candidate genes have been involved in the plethora of cellular functions related to response to DNA damage, cell cycle, cell growth, etc. The PPI network was retrieved through GeneMANIA and STRING server for *ATM*, *CHEK2*, *PALB2*, and *XRCC2*, as depicted in Figures 7A–H. These networks show that the candidate genes are involved intricate networks related to cellular repair mechanisms.

In the current study, we have used the NG-WES for identifying the frequency of mutations and mutational landscape. It is the first report on characterizing the mutational landscape of BC patients from Khyber Pakhtunkhwa in a specifically enrolled cohort with Pashtun ethnicity. Furthermore, we have assessed the mutational spectrum in the context of their association with various hormonal, non-hormonal, and clinicopathologic features.

Ataxia-telangiectasia mutated (*ATM*) is an oncosuppressor gene which codes for a 350-KDa protein with 3,056 amino acids that perform the function of activating DNA repair pathways. The *ATM* gene plays different roles in cellular processes like energy production, oxidative homeostasis, telomere maintenance, chromatin remodeling, and genomic integrity, which are considered crucial processes in the context of cancer progression (Phan and Rezaeian, 2021). *ATM* mutations have been detected in up to 40% of BC patients (Stucci et al., 2021). *ATM* mutations are frequently reported in the development of BC. Approximately 5% solid tumors are reported with *ATM* aberrations (Stucci et al., 2021). Structurally, the *ATM* gene comprises four major domains including the spiral domain (1–1160), pincer domain (1161–1890); FAT domain (1903–2612), and kinase domain (2618–3056) (Ueno et al., 2022). We reported three mutations in the *ATM* spiral domain (residues 1–1161), which plays a potential role in the binding adaptors, regulators, and substrates (Baretić et al., 2017). Six mutations are reported in the pincer domain of *ATM*. We have reported one mutation *ATM p.G2023R* in the FAT domain, and such mutations have the potential to increase the risk of breast cancer (Cavaciuti et al., 2005).

In our results, various *ATM* mutations have been reported previously. One of them, i.e., *ATM p.D1853N*, has been previously reported; however, it is considered of least predictive value and has a weak association with developing breast cancer (Moslemi et al., 2021; Schrauder et al., 2008). *ATM p.G2023R* has been previously reported in breast cancer patients from Brazil; however, we obtained the same mutation on exon 41, whereas, in the Brazilian cohort, the same mutation was reported on exon 42 (Mangone et al., 2015). *ATM p.S978P* has been reported to have implications in pancreatic cancer (Ding et al., 2021).

*CHEK2* is well-characterized for playing an important role in cell cycle regulation and apoptosis after the cells are exposed to DNA damage. *CHEK2* is activated by *ATM*, and its activation triggers a downstream cascade of cellular events that ensure genomic integrity and DNA repair (Boonen et al., 2022). Mutations in *CHEK2* have been reported to have a moderate risk of BC. We have obtained three mutations in *CHEK2*, i.e., *CHEK2 p.W114L*, *p.R180C*, and *p.M314I,* in which the first two mutations are located in the region referred to as forkhead-associated domain (FHA) located approximately from 113 to 180 residues, whereas *CHEK2 p.M314I* is located in the kinase domain that further activates the downstream effector proteins (Stubbins et al., 2022). The FHA domain is responsible for *CHEK2* dimerization in a phosphorylation-dependent manner (Durocher and Jackson, 2002), which is considered important for full activation of *CHEK2* by trans-phosphorylation within the kinase domain (Kleibl et al., 2008). Mutations in these regions can compromise the functional properties of *CHEK2*, eventually, leading to the BC. The *CHEK2* mutations have previously been reported in breast cancer. *CHEK2 p.R180C* has been reported in breast cancer of familial nature in German and Jewish ethnicity (Dufault et al., 2004). *CHEK2 p.R180C* mutations have also been reported in Chinese and Malay (Mohamad et al., 2015).

*PALB2* (partner and localizer of *BRCA2*) is considered a high-risk gene in breast cancer, and it encodes for a protein with tumor suppressor activity. Its pathogenic variants have the risk of 30%–60% of developing BC in women (Nepomuceno et al., 2021). The *PALB2*-encoded protein binds and colocalizes with *BRCA2* and forms a *BRCA1*–*PALB2*–*BRCA2* complex. The major function of *PALB2* is to associate with *BRCA2* for maintaining the genomic integrity and preventing the accumulation of DNA mutations (Evans and Longo, 2014). Our results revealed three *PALB2* mutations, i.e., *PALB2 p.Q559R*, *p.I1013K*, and *p.T1029S* in the enrolled cohort. *PALB2 p.Q559R* was reported previously in the Italian cohort of breast cancer patients with comparatively higher penetrance (Silvestri et al., 2010), which correlates with our findings for the Pashtun ethnicity.

*XRCC2* (X-ray repair cross-complementing) is another gene with cancer predisposition. The *XRCC2* protein product plays a role in repairing the DSBs through homologous recombination repair (HRR) and apoptosis. A defective homologous recombination eventually leads to cancer progression. *XRCC2* is a *RAD51* paralogue and considered essential in the HRR process. Mutations in *XRCC2* compromise the DNA repair mechanisms and increase susceptibility to cancers (He et al., 2014). We have reported one mutation *XRCC2 p.R188H*, which is already well-established to have an association with the cancer progression (García-Closas et al., 2006). *XRCC2 p.R188H* was previously reported from India (Datkhile et al., 2023).

The molecular dynamic simulation results revealed differences in the radius of gyration and root mean square deviation. It has been reported that the SNVs can perturb the nature of the proteins or its segments and may cause shift in the equilibrium between different conformations, destabilizes the protein, or modifies the conformational dynamics. From the results of the MDS, it was clearly evident that for all the IS mutations, the radius of gyration for the mutant proteins has increased, as compared to their wild type. This signifies that the mutant proteins have a perturbed unfolded nature, as compared to the wild types.

The RMSD trajectories were used to compare the differences in the backbone from initial conformation to its final conformation. The protein stability in the context of its conformation can be deduced from the deviations produced during the simulation time. Larger deviations are synonymous with the perturbed structure of the protein (Aier et al., 2016). We observed major deviations in the RMSD comparison of *XRCC p.R188H*, as compared to the others, where relatively smaller deviations were observed. These results of the MDS simulations complemented the *in silico* predictions using SAAFEQ-SEQ, which concluded the destabilizing effect for all of the obtained SNVs including the interacting site mutations.

We docked the proteins and their mutants with two compounds of interest (DB11979 and LTS0102038). The effect of mutations on binding affinity varied depending on the protein and the compound. Binding residues also got altered, while some were conserved in binding in normal or mutated states. Mutations can have varying effects on affinity due to the alteration of involved binding residues. Understanding these interactions is essential for drug design and understanding the impact of genetic mutations on protein function. We propose that FDA-approved drug repurposing, nutraceutical, and natural product screening should be attempted at a wider scale against the proteins and their mutants of interest. We also propose that the *in silico* impact should be further explored by complementary *in silico*, *in vitro*, and *in vivo* assays.

## Conclusion

The present study pioneered the acquisition of the mutational landscape of the breast cancer susceptibility genes (*ATM*, *CHEK2*, *PALB2*, and *XRCC2*) using next-generation whole-exome sequencing from paraffin-fixed FFPE tissue blocks obtained from the breast cancer patients of Pashtun ethnicity. The acquired sequence data were studied in the context of sociogenetic and clinicopathologic features. We found that the *ATM* was frequently mutated, as compared to others. Out of the total 18 mutations (14 somatic and 4 germline), 8 mutations were identified as novel. Seventeen mutations were nonsynonymous SNVs. SIFT, PolyPhen-2, and MutationTaster databases were used to examine pathogenicity and tolerability. *PALB2 p.Q559R* was the most prevalent mutation among the patients that can be further studied in larger cohorts for biomarker implications. The molecular dynamics simulation study revealed that the SNVs contributed to a perturbed protein-folding behavior. *CHEK2 p.R180C* was found only in PR+, and the remaining *CHEK2 p.M314I* and *p.W114L* were only found in PR-. Molecular docking results showed that mutations altered the drug interactions.

Less sample size and enrollments forming a single region are the limitations to the study.

## Data Availability

The datasets presented in this study can be found in online repositories. The names of the repository/repositories and accession number(s) can be found at: https://www.ncbi.nlm.nih.gov/, PRJNA941166.
