# Supplementary material for: Clinico-genomic findings, molecular docking, and mutational spectrum in an understudied population with breast cancer patients from KP, Pakistan
Source: Front Genet. 2024 May 9;15:1383284. doi: 10.3389/fgene.2024.1383284 (PMC11111998; doi:10.3389/fgene.2024.1383284)
Supplement: Supplementary file 1 [file Table1.DOCX]

| **Patient** codes | **Gene** | **Mutation Type** | **Reported** | **Status** | **Location (Exon)** | **Mutations** | **Mutation**  **Label** | **gnomAD_exome_ALL** | **dbSNP** | **ClinVar_SIG** | **CADD**  **Score** | Mutation  **Assessor** | **LRT** | **FATHMM** | **PROVEAN** | **MetaSVM** | **MetaLR** | **M.CAP** |  |
| --- | --- | --- | --- | --- | --- | --- | --- | --- | --- | --- | --- | --- | --- | --- | --- | --- | --- | --- | --- |
| ***ATM*** | | | | | | | | | | | | | | | | | | | |
| BCR 2T, 4T, 25T, 27T | *ATM* | nonsynonymous SNV | dbSNP/Cosmic | Somatic | exon37 | NM_000051.4:*c.5557G>A* | *p.D1853N* | 0.1146 | rs1801516 | other\|Benign\|Likely benign | 23.2 | L | D | T | N | T | T | . |  |
| BCR 22T | *ATM* | nonsynonymous SNV | dbSNP/Cosmic | Somatic | exon41 | NM_000051.4:*c.6067G>A* | *p.G2023R* | 0.0015 | rs11212587 | Likely benign\|other\|Likely benign | 31 | M | D | T | D | T | T | D |  |
| BCR 24T | *ATM* | nonsynonymous SNV | dbSNP/Cosmic | Somatic | exon20 | NM_000051.4:*c.2932T>C* | *p.S978P* | 0.001 | rs139552233 | Uncertain significance\|Benign\|other | 26.8 | M | D | T | D | D | D | D |  |
| BCR 85T | *ATM* | frameshift deletion | Novel | Somatic | exon22 | NM_000051.4:*c.3209delT* | *p.V1070Efs*38* | . | . | . | . |  | . | . | . | . | . | . |  |
| BCR 85T | *ATM* | nonsynonymous SNV | Novel | Somatic | exon35 | NM_000051.4:*c.5280G>T* | *p.M1760I* | . | . | . | 18.77 | M | N | T | N | T | T | D |  |
| BCR 114T | *ATM* | nonsynonymous SNV | Novel | Somatic | exon7 | NM_000051.4:*c.796T>C* | *p.W266R* | . | . | . | 26.2 | M | D | T | D | T | T | D |  |
| BCR 114 T/N | *ATM* | nonsynonymous SNV | dbSNP/Cosmic | Germline | exon37 | NM_000051.4:*c.5630T>C* | *p.F1877S* | 4.51E-05 | rs202028401 | Uncertain significance\|Uncertain significance | 0.032 | N | N | T | N | T | T | T |  |
| BCR 116T | *ATM* | nonsynonymous SNV | Novel | Somatic | exon28 | NM_000051.4:*c.4209C>A* | *p.S1403R* | . | . | . | 23.5 | M | D | T | N | T | T | D |  |
| BCR 116T | *ATM* | nonsynonymous SNV | Novel | Somatic | exon31 | NM_000051.4:*c.4702C>A* | *p.H1568N* | . | . | . | 20.2 | L | N | T | N | T | T | T |  |
| BCR 118T | *ATM* | nonsynonymous SNV | dbSNP/Cosmic | Somatic | exon28 | NM_000051.4:*c.4138C>T* | *p.H1380Y* | 0.0121 | rs3092856 | Benign\|Benign\|other | 6.964 | N | N | T | N | T | T | . |  |
| BCR 120T/N | *ATM* | nonsynonymous SNV | dbSNP/Cosmic | Germline | exon22 | NM_000051.4: *c3175G>T* | *p.A1059S* | 4.06E-06 | rs370282831 | Uncertain significance\|Uncertain significance\|Uncertain significance\|Uncertain significance | 12.75 | M | N | T | N | T | T | D |  |
| ***CHEK2*** | | | | | | | | | | | | | | | | | | | |
| BCR 4T | *CHEK2* | nonsynonymous SNV | Cosmic | Somatic | exon4 | NM_007194:*c.538C>T* | *p.R180C* | 0.001 | rs77130927 | Likely benign\|other\|Uncertain significance | 23.2 | M | D | D | D | D | D | D |  |
| BCR 120T | *CHEK2* | nonsynonymous SNV | Novel | Somatic | exon10 | NM_001349956: *c.942G>T* | *p.M314I* | . | . | . | 33 | N | D | T | D | T | T | D |  |
| BCR 120T | *CHEK2* | nonsynonymous SNV | Clinvar | Somatic | exon3 | NM_001349956:*c.341G>T* | *p.W114L* | . | . | . | 21.3 | N | N | D | N | T | T | D |  |
| ***PALB2*** | | | | | | | | | | | | | | | | | | | |
| BCR 4T, 24T, 27T, 85T, 90T/N, 116T/N | *PALB2* | nonsynonymous SNV | Cosmic | Germline | exon4 | NM_024675.4:*c.1676A>G* | *p.Q559R* | 0.1043 | rs152451 | other\|Benign\|Benign\|Benign | 0.001 | N | N | T | N | T | T | . |  |
| BCR 85T | *PALB2* | nonsynonymous SNV | Novel | Somatic | exon10 | NM_024675.4:*c.3086C>G* | *p.T1029S* | . | . | . | 23.7 | L | D | T | D | T | T | T |  |
| BCR 118T | *PALB2* | nonsynonymous SNV | Novel | Somatic | exon10 | NM_024675.4:*c.3038T>A* | *p.I1013K* | . | . | . | 23.9 | L | N | T | D | T | T | D |  |
| ***XRCC2*** | | | | | | | | | | | | | | | | | | | |
| **BCR 82T/N, 90T/N, 116T/N, 117T/N,**  **120T/N** | *XRCC2* | nonsynonymous SNV | Cosmic | Germline | exon3 | NM_005431.2:*c.563G>A* | *p.R188H* | 0.0639 | rs3218536 | . | 15.95 | N | N | T | N | T | T | . |  |

Table S-2: Mutation predictions of various databases

**Caption**

**CADD Score: “Combined Annotation Dependent Depletion”; Mutation Assessor Pred:”H: high; M: medium; L: low; N: neutral. H/M means functional and L/N means non-functional”; FATHMM Pred: “D: Deleterious; T: Tolerated”; MetaSVM Pred: “D: Deleterious; T: Tolerated”; MetaLR Pred: D: Deleterious; T: Tolerated; LRT Pred: D: “Deleterious; N: Neutral; U: Unknown”; PROVEAN Pred: “D: Deleterious; N: Neutral”; M. CAP Pred: “D: Deleterious; T: Tolerated”; dbSNP: “Single Nucleotide Polymorphism Database”**

Note: BCR (patient enrollment code “Breast Cancer Research”); “T” for Tumor FFPE block; “N” for Normal FFPE block
