# Supplementary material for: Clinico-genomic findings, molecular docking, and mutational spectrum in an understudied population with breast cancer patients from KP, Pakistan
Source: Front Genet. 2024 May 9;15:1383284. doi: 10.3389/fgene.2024.1383284 (PMC11111998; doi:10.3389/fgene.2024.1383284)
Supplement: Supplementary file 2 [file Table2.DOCX]

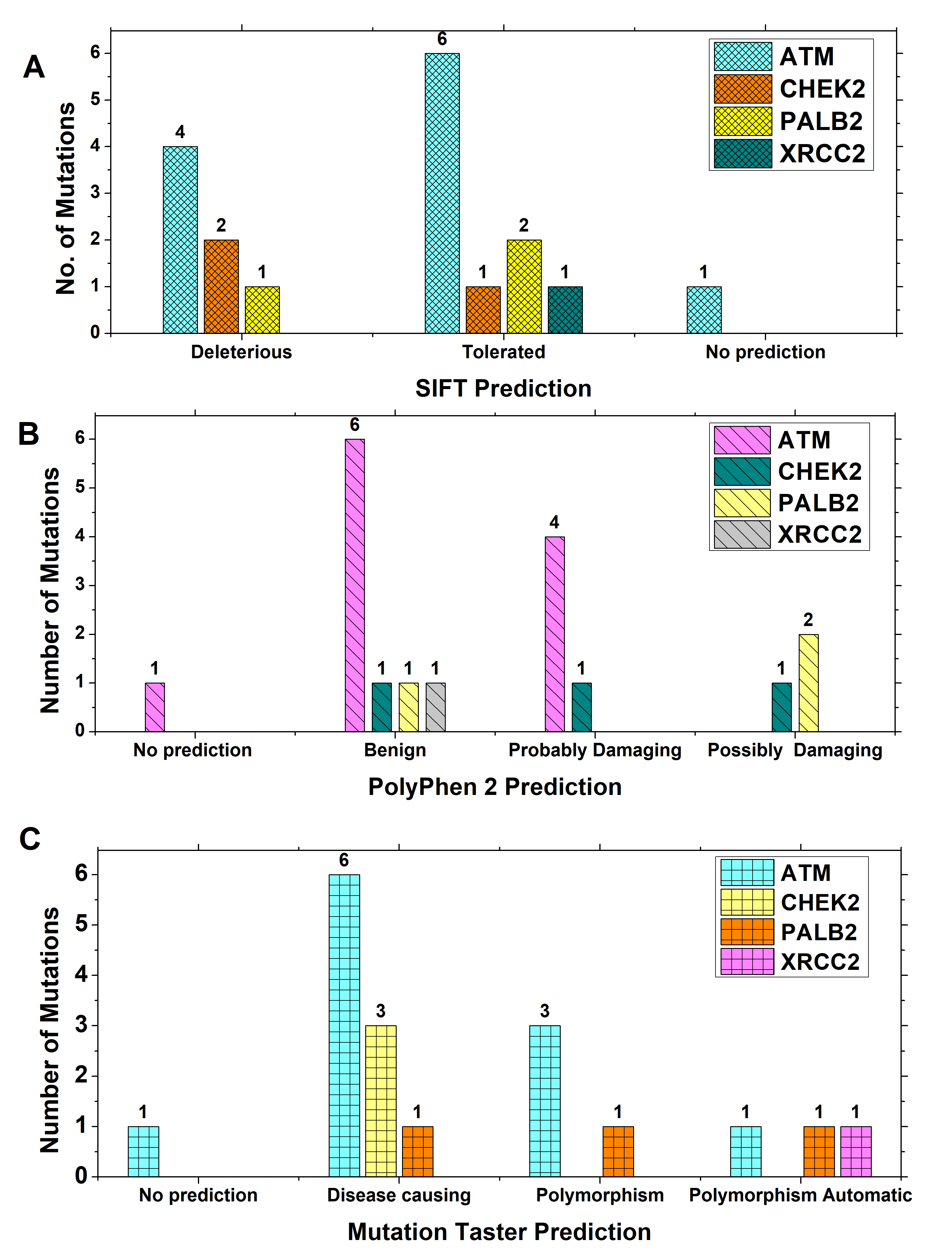


**Figure 4-S: Gene wise mutation predictions from different databases in the enrolled cohort; (A) SIFT Prediction; (B) PolyPhen 2 Prediction; (C) Mutation Taster Prediction**


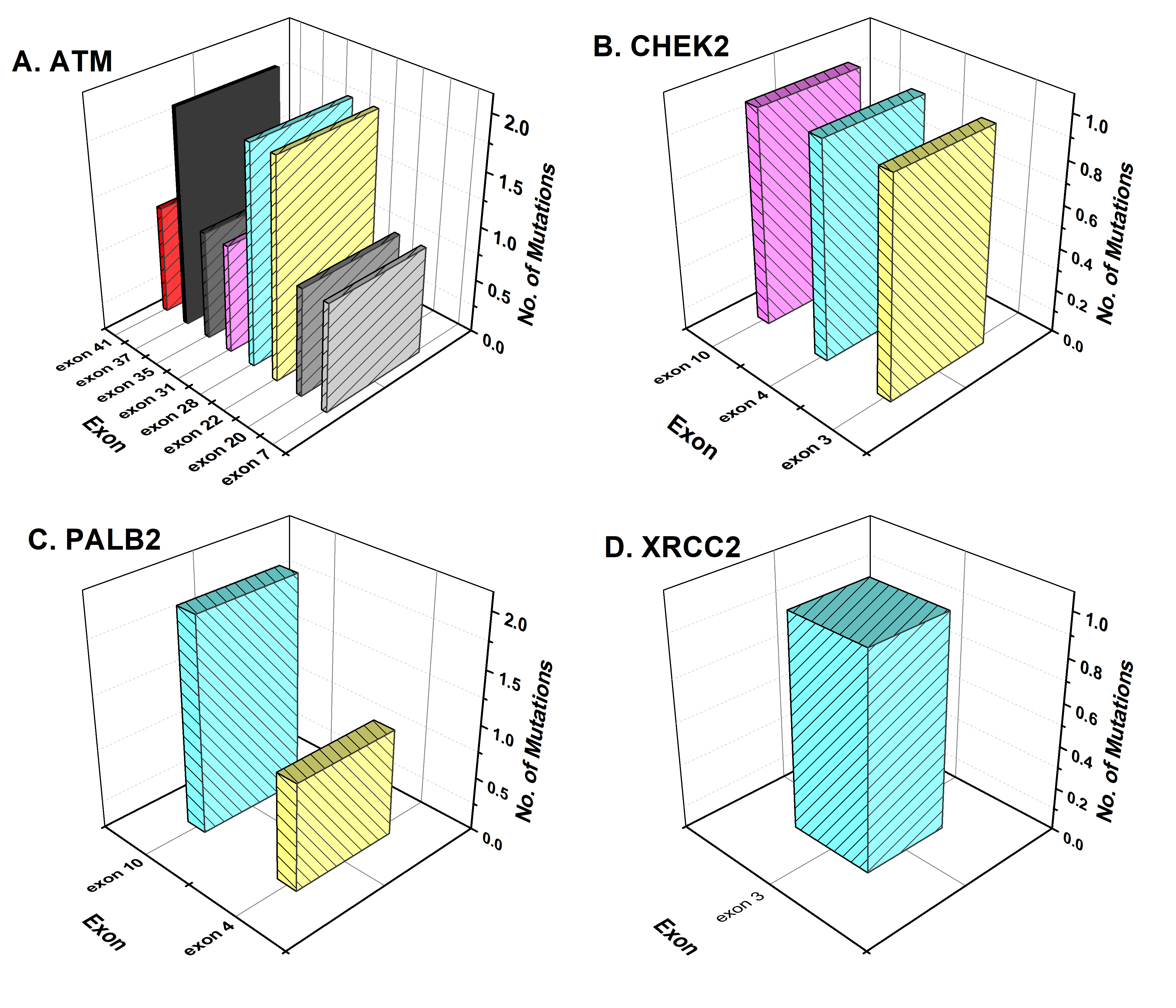


**Figure 5-S: Mutation distribution on exons; (A) *ATM*; (B) *CHEK2*; (C) *PALB2*; (D) *XRCC2***


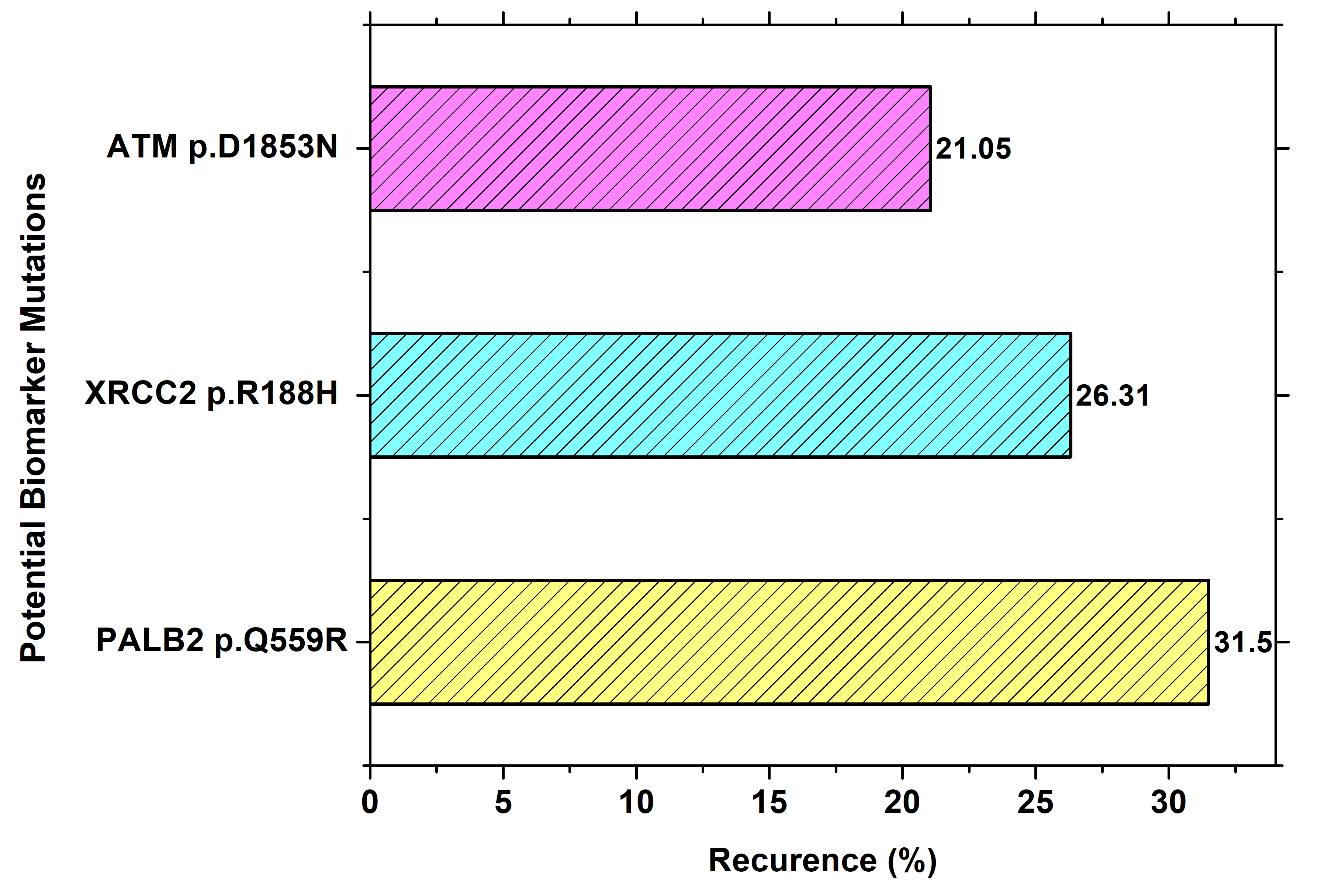


**Figure 7-S: Mutations with biomarker potential**


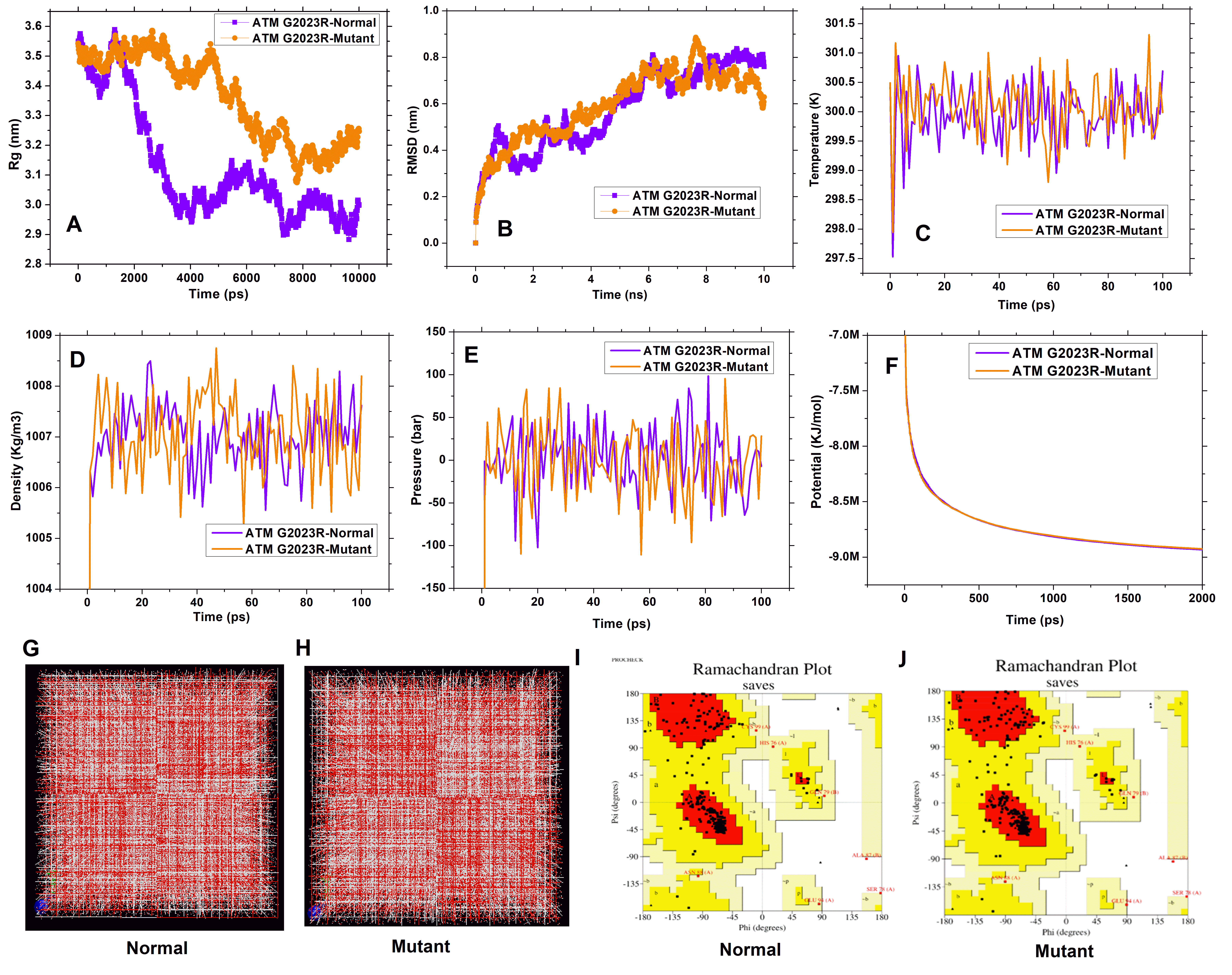


**Figure 9-S: Results of the molecular dynamic simulation of normal and mutant *ATM p.G2023R* using Gromacs (A-H) and their Ramachandran plots (I-J)**


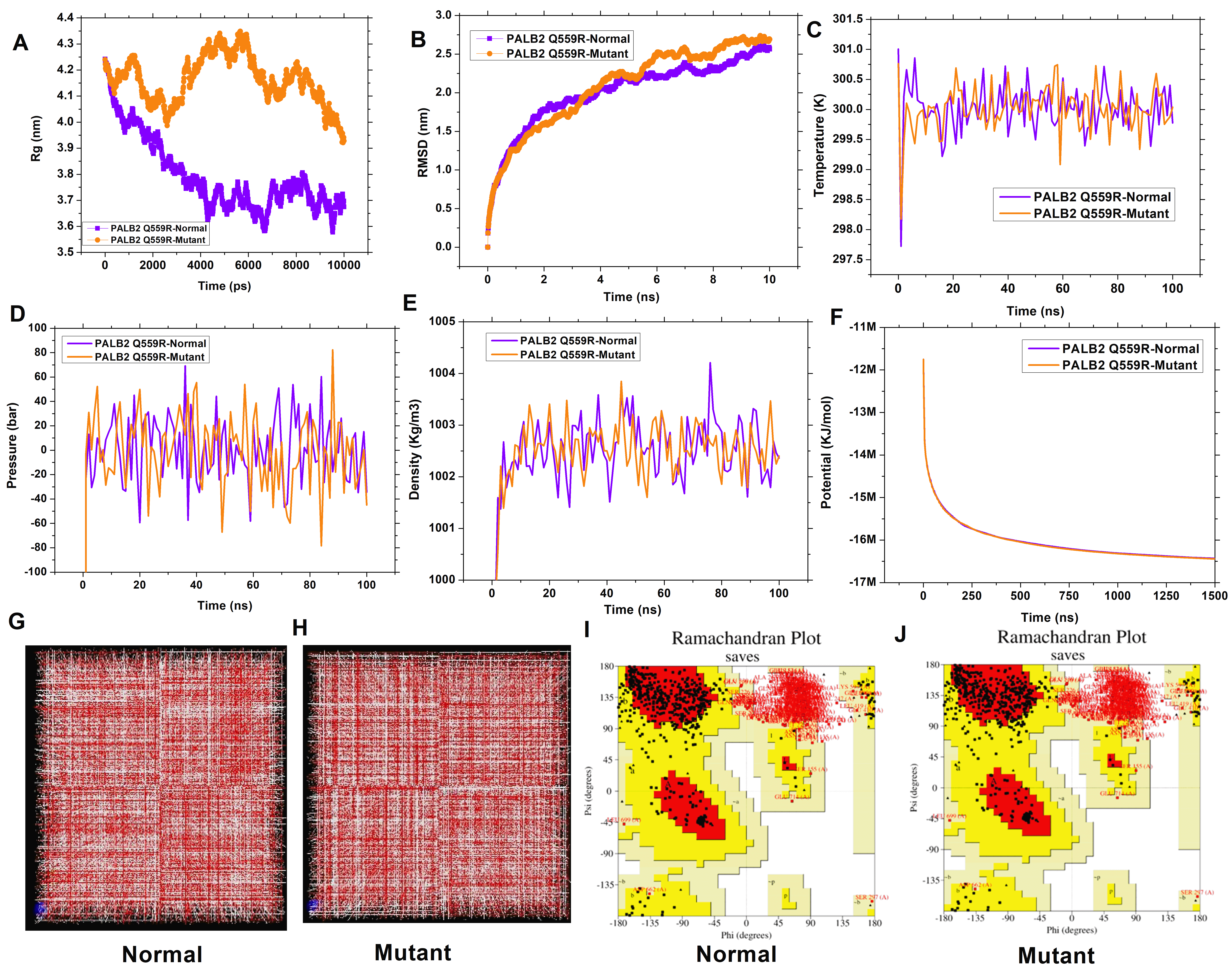


**Figure 10-S: Results of the molecular dynamic simulation of normal and mutant *PALB2 p.Q559R* using Gromacs (A-H) and their Ramachandran plots (I-J)**


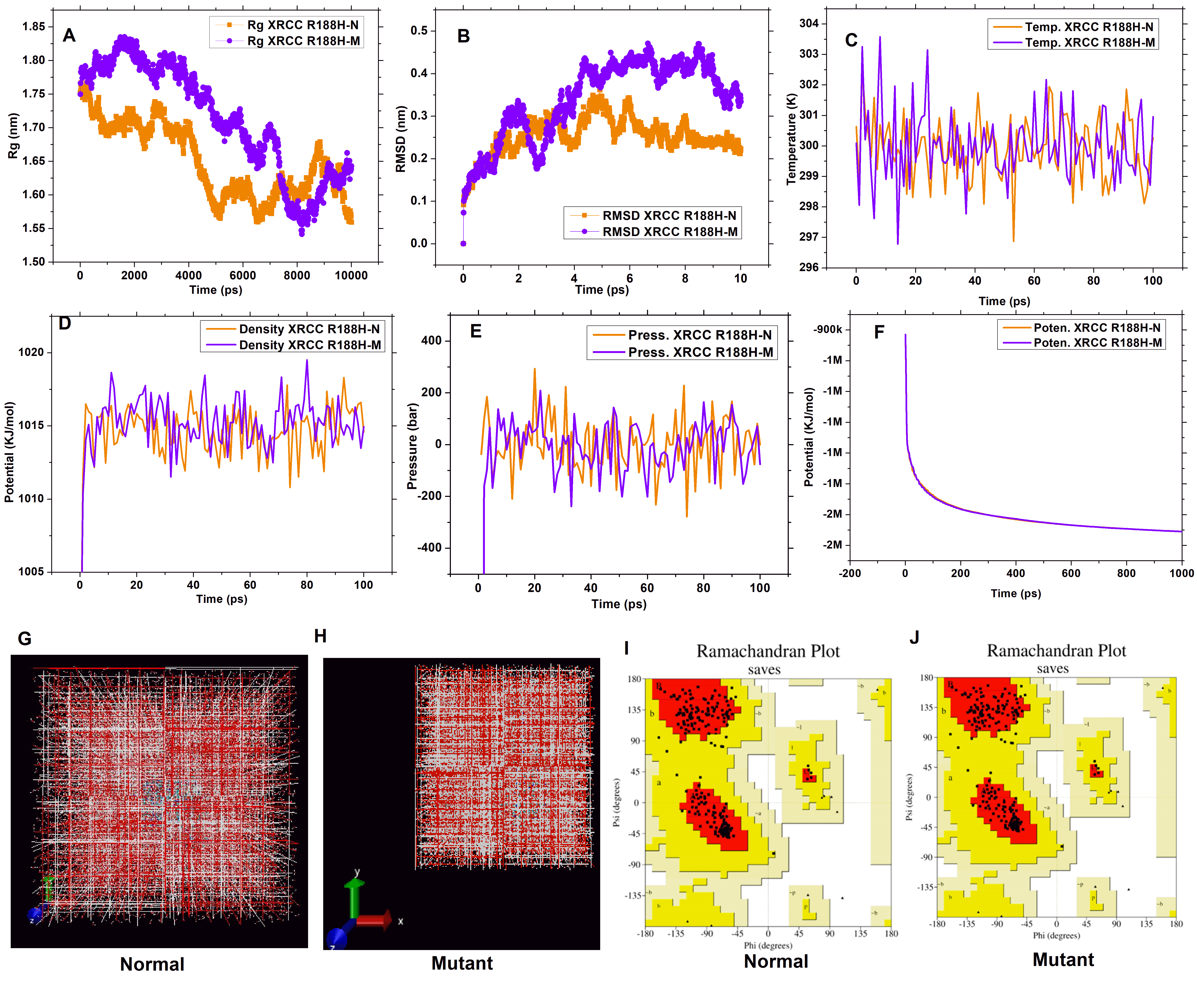


**Figure 11-S: Results of the molecular dynamic simulation of normal and mutant *XRCC2 p.R188H* using Gromacs (A-H) and their Ramachandran plots (I-J)**


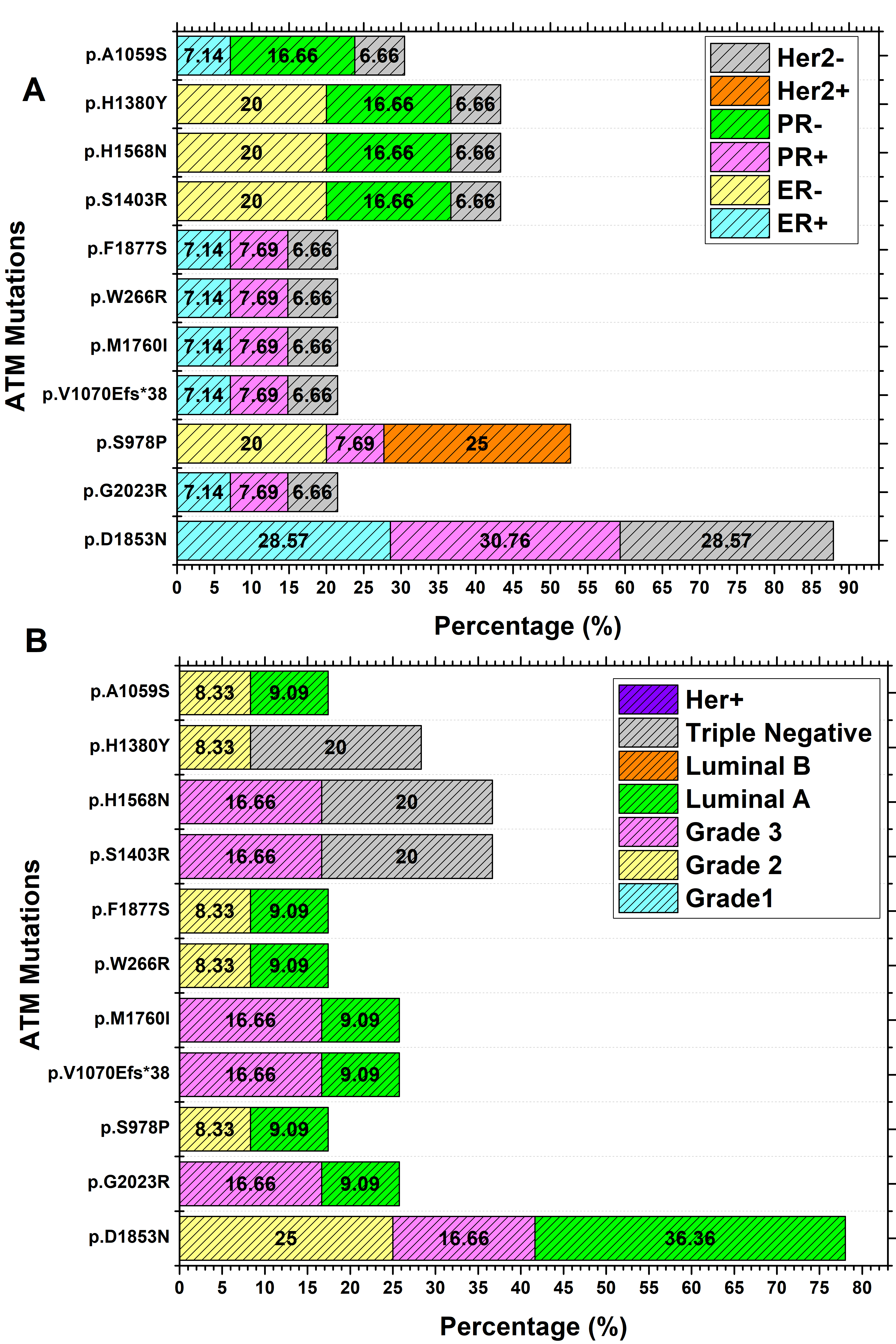


**Figure 12-S: Association of *ATM* mutations with (A) Immunohistochemical markers and (B) Molecular subtypes and tumor grades**


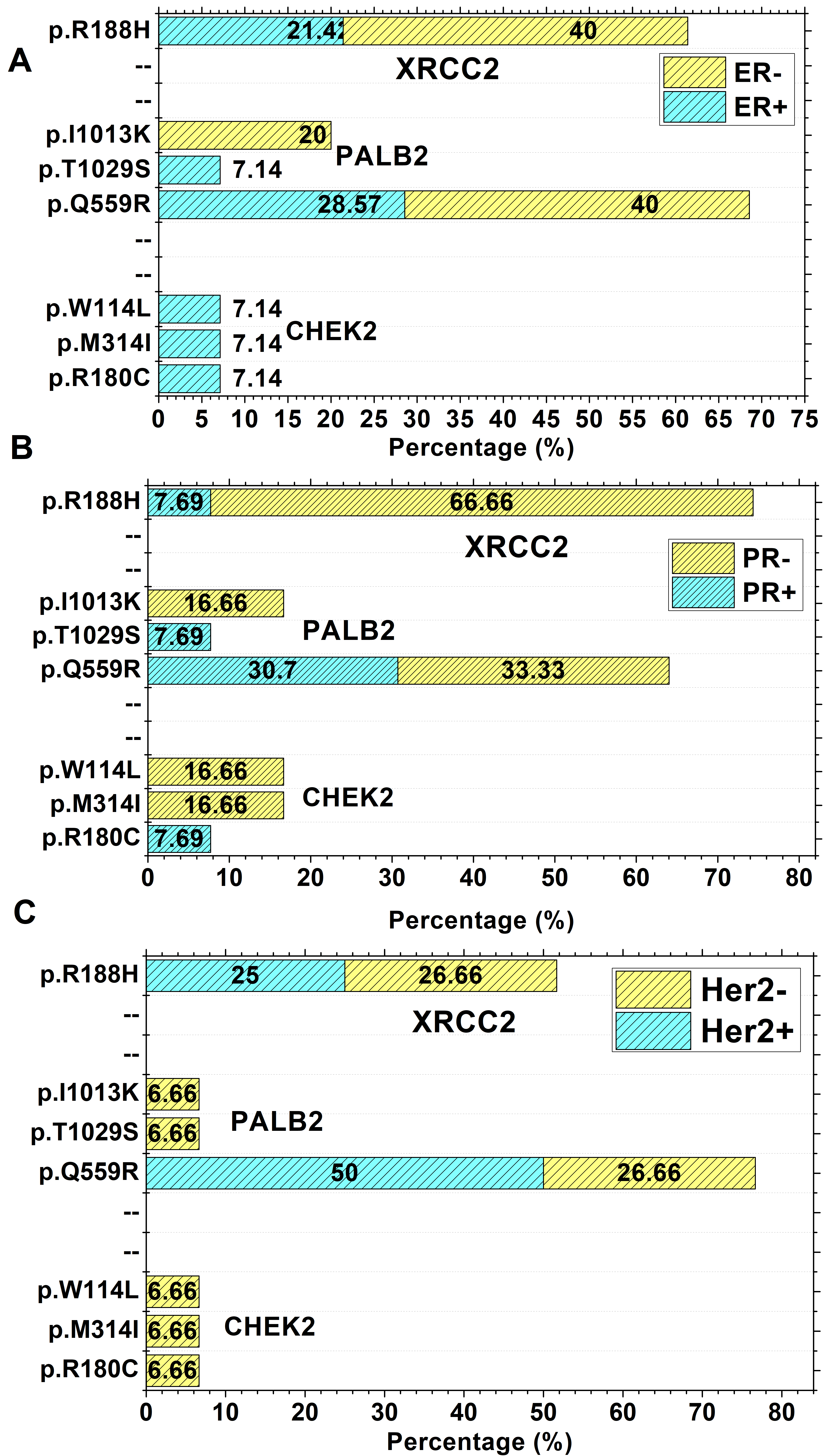


**Figure 13-S: Clinicopathological association of *CHEK2*, *PALB2*, *XRCC2* with immunohistochemical markers**


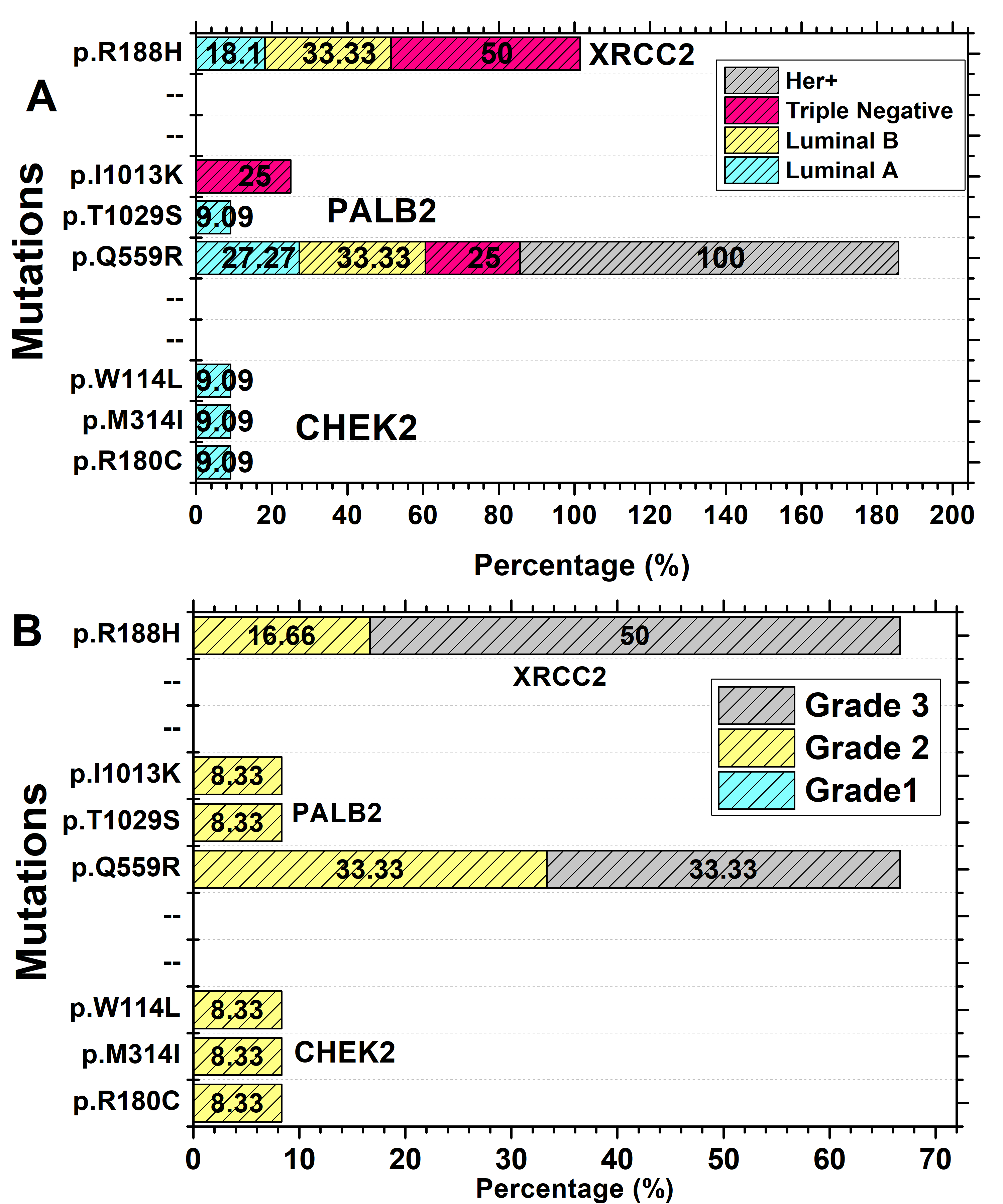


**Figure 14-S: Association of *CHEK2*, *PALB2*, *XRCC2* with (A) Molecular subtypes and (B) tumor grades**
